# Overexpression of TBX3 suppresses tumorigenesis in experimental and human cholangiocarcinoma

**DOI:** 10.1038/s41419-024-06839-8

**Published:** 2024-06-22

**Authors:** Shanshan Deng, Xinjun Lu, Xue Wang, Binyong Liang, Hongwei Xu, Doris Yang, Guofei Cui, Andrew Yonemura, Honor Paine, Yi Zhou, Yi Zhang, Maria Maddalena Simile, Francesco Urigo, Matthias Evert, Diego F. Calvisi, Benjamin L. Green, Xin Chen

**Affiliations:** 1 Cancer Biology Program, University of Hawai’i Cancer Center, University of Hawai’i, Honolulu, HI USA; 2 Department of Bioengineering and Therapeutic Sciences and Liver Center, University of California, San Francisco, CA USA; 3 Department of Biliary-Pancreatic Surgery, Sun Yat-sen Memorial Hospital, Sun Yat-sen University, Guangzhou, China; 4 School of Pharmacy and Bioengineering, Chongqing University of Technology, 400054 Chongqing, China; 5 Department of Medicine, Surgery, and Pharmacy, Division of Experimental Pathology and Oncology, University of Sassari, 07100 Sassari, Italy; 6 Institute of Pathology, University of Regensburg, Regensburg, Germany

**Keywords:** Tumour-suppressor proteins, Targeted therapies

## Abstract

TBX3 behaves as a tumor suppressor or oncoprotein across cancer. However, TBX3 function remains undetermined in intrahepatic cholangiocarcinoma (iCCA), a deadly primary liver malignancy with few systemic treatment options. This study sought to investigate the impact of TBX3 on iCCA. We found that overexpression of TBX3 strongly inhibited human iCCA cell growth. In the *Akt/FBXW7ΔF* mouse iCCA model, overexpression of *Tbx3* reduced cholangiocarcinogenesis in vivo, while inducible genetic knockout of *Tbx3* accelerated iCCA growth. RNA-seq identified *MAD2L1* as a downregulated gene in TBX3-overexpressing cells, and ChIP confirmed that TBX3 binds to the MAD2L1 promoter. CRISPR-mediated knockdown of *Mad2l1* significantly reduced the growth of two iCCA models in vivo. Finally, we found that TBX3 expression is upregulated in ~20% of human iCCA samples, and its high expression is associated with less proliferation and better survival. MAD2L1 expression is upregulated in most human iCCA samples and negatively correlated with TBX3 expression. Altogether, our findings suggest that overexpression of TBX3 suppresses CCA progression via repressing MAD2L1 expression.

## Introduction

Cholangiocarcinoma (CCA), the second most common primary liver cancer after hepatocellular carcinoma (HCC), accounts for approximately 15% of all primary hepatic malignancies and is responsible for a high mortality rate [1–3]. CCAs are divided into three subtypes according to the anatomical site of origin: intrahepatic cholangiocarcinoma (iCCA), perihilar cholangiocarcinoma (pCCA), and distal cholangiocarcinoma (dCCA) [4]. Unlike perihilar cholangiocarcinoma (pCCA) or distal cholangiocarcinoma (dCCA), developing in the extrahepatic bile ducts, intrahepatic cholangiocarcinoma (iCCA) occurs above the second-order bile ducts, within the hepatic parenchyma [5]. Although the incidence of iCCA is lower than that of extrahepatic cholangiocarcinoma (eCCA), the global incidence of iCCA is experiencing a significantly faster growth rate than eCCA [6–8].

For iCCA, surgery is the primary treatment option, but up to two-thirds of patients experience disease recurrence after surgery due to the advanced stages of the disease when diagnosed [9, 10]. Despite surgical resection, the median survival time and 5-year overall survival rate of patients with iCCA remain relatively low [10]. Systemic chemotherapy and targeted therapies are available for unresectable iCCAs [11, 12]. First-line treatment consists of gemcitabine plus cisplatin, and adding the immune checkpoint inhibitor durvalumab is currently recommended [13]. However, with time, most patients develop resistance to treatment, leading to tumor progression. Recent studies revealed actionable genetic alterations in patients with iCCA, increasing interest among researchers in precision medicine approaches [13–15]. Although targeted therapies demonstrated enhanced efficacy against particular iCCA subtypes bearing specific molecular alterations, these cases represent the minority, and the benefits of the treatments are generally temporary. Overall, systemic treatment options have limited success partially due to the incomplete understanding of iCCA pathogenesis and biology. Thus, additional efforts are required to improve the outcomes for patients with iCCA, and further exploration of critical molecular pathways and targets of iCCA is needed.

TBX3 is a member of the T-box gene family and functions as a transcription factor [16]. TBX3 is the germline genetic variant associated with the ulnar-mammary syndrome, a rare pleiotropic disorder with an autosomal dominant inheritance pattern and incomplete penetrance affecting upper limb development and ulnar and mammary apocrine sweat glands [17]. Recent studies revealed TBX3 overexpression in diverse cancer types, which facilitates tumor development and progression [18–20]. However, TBX3 acts as a tumor suppressor in other tumor entities, including β-catenin-driven HCC [18, 21, 22]. Given the ongoing debate regarding the role of TBX3 in liver cancer and the limited knowledge of its specific involvement in iCCA, our study aimed to investigate the expression pattern of TBX3 and elucidate its functional role in iCCA.

## Methods

### Constructs and reagents

The plasmids used for cell transfection and Crispr-Cas9 for gene knockout include *pLenti-HA-puro-TBX3 (human), pLenti-HA-puro-MAD2L1 (human)*, and *H138-puro-sgMAD2L1* (human). The plasmids used for mouse injection in this study consist of *pT3* (vector), *pT3-EF1α-HA-myr-Akt* (human), *pT3-EF1α-HA-Fbxw7ΔF* (human), *pT3-EF1α-NICD* (human), *pT3-EF1α-HA-TBX3* (human), *pCMV*, *pCMV-Cre*, *pX330-sgEGFP*, *pX330-sgMad2l1*, and pCMV-Sleeping Beauty transposase. All the plasmids used in cell transfection or mouse injection were generated by inserting the corresponding human cDNA into pLenti-puro or pT3-EF1α vector or gRNA into H138 or pX330 vector via the Gateway cloning system (Invitrogen), as described previously [21, 23]. Plasmids were purified using the Endotoxin-free Plasmid Maxiprep Kit (Sigma-Aldrich, MO).

### TCGA/NCI data analysis

The relative mRNA level of *TBX3* in human intrahepatic cholangiocarcinoma relative to normal liver tissues from The Cancer Genome Atlas Cholangiocarcinoma Consortium (TCGA) [24] and National Cancer Institute (NCI) [25] datasets were plotted using the GraphPad Prism 7 software.

### Cell culture and in vitro studies

RBE, KKU-156, and HUCCT1 human iCCA cell lines were donated by Dr. Gregory J. Gores (Mayo Clinic, Rochester, MN). The human embryonic kidney cell line HEK-293FT was purchased from the American Type Culture Collection (Manassas, VA). Cells were cultured in Dulbecco’s modified Eagle medium (Corning, NY) supplemented with 10% fetal bovine serum (FBS, Sigma-Aldrich) and 1% penicillin-streptomycin (PS) solution (Sigma-Aldrich), and maintained at 37 °C in a 5% CO_2_ incubator. For lentivirus packaging and cell transfection, we used HEK-293FT cells in earlier passages for lentivirus packaging and production. HEK-293FT cells were cultured in PS-free DMEM medium for 2 days before lentivirus packaging. We seeded HEK-293FT cells in 6cm-plates, and the cells were incubated at 37 °C, 5% CO_2,_ until reaching 60-70% confluence. Subsequently, after 5 min of incubation of 7.5 μL of Lipofectamine 2000 reagent (Invitrogen, MA) in 125 μL of Opti-MEM medium, the plasmid mixture (2.3 μg psPAX2 + 0.7 μg pMD2.G + 3 μg Plenti-Gene or H138-sgGene) in 125 μL Opti-MEM medium was added to the Lipofectamine 2000 reagent mixture and incubated at room temperature for another 30 min. Next, 250 μL of viral packaging mixture was added to HEK-293FT cells containing 2 mL of PS-free DMEM medium and incubated overnight in the incubator. The next day, the medium containing the viral packaging mixture was replaced with 1 mL of PS-free DMEM medium for virus collection. After 8 hours, the lentivirus-containing medium was filtered and added to RBE, KKU-156, and HUCCT1 cells with a cell confluence of 70%. At the same time, the HEK-293FT cells were replaced with a new 1 mL PS-free DMEM medium for virus collection. This step was repeated twice. Afterward, the old medium from RBE, KKU-156, and HUCCT1 cells was replaced by a full-growth medium for overnight recovery. The next day, cells were selected using a puromycin-containing medium (2 μg/mL for RBE, 1.5 μg/mL for KKU-156, and 1.0 μg/mL for HUCCT1) to select for cells with stable expression of the target gene and puromycin resistance. PCR assay and Sanger sequencing were conducted to determine the knockout efficiency of selected cells. The sequence of sgMAD2L1 is reported in Supplementary Table 1.

For colony formation, iCCA parental cell lines, including RBE, KKU-156, and HUCCT1 cells and their corresponding gene-edited cells, were seeded into 6-well plates (1000 cells/well) in triplicate. The fresh medium was replaced every three days. One week later, cells were fixed with methanol and stained with 0.5% crystal violet. Colonies were imaged and quantified using the ImageJ software (National Institutes of Health, MD).

### Protein extraction and western blot analysis

Human cells or mouse liver tissues were homogenized in the Mammalian Protein Extraction Reagent (Thermo Fisher Scientific, #78501) containing the Halt protease inhibitor cocktail (Thermo Fisher Scientific, #78429). Subsequently, the protein concentration in the supernatant was detected with the BCA Protein Assay Kit. Denatured proteins were separated in SDS-PAGE gels and transferred to nitrocellulose membranes (Bio-Rad Laboratories). Membranes were blocked in 5% skim milk for 1 h at room temperature, then incubated with primary antibodies overnight at 4 °C. Primary antibodies included rabbit anti-GAPDH (1:10000, Cell Signaling Technology, #5174), rabbit anti-TBX3 (1:250, Invitrogen, #42-4800), rabbit anti-HA-Tag (1:1000, Cell Signaling Technology, #3724) and rabbit anti-MAD2L1 (1:1000, Proteintech Group, Inc., #10337-1-AP). The membranes were subsequently incubated with goat anti-rabbit-IgG-HRP (#7074, 1:1,000, Cell Signaling Technology) secondary antibodies for 1 h at room temperature. After appropriate washing, the membranes were developed with the ECL substrate (Bio-Rad, #170-5061) and visualized using X-ray films.

### RNA extraction and quantitative real-time PCR

Total mRNA from cells and mouse tissues was extracted and purified using the Zymo RNA Miniprep Kit (Zymo Research, CA) according to the manufacturer’s instructions. The concentration of mRNA was determined using a NanoDrop™ 2000 spectrophotometer (Thermo Scientific™, MA). RNA was then reverse transcribed using the Prime Script Reverse Transcription Kit (Thermo Scientific™). After reverse transcription, the cDNA was diluted 1:10 with sterile water and stored at −20 °C. Quantitative real-time PCR (RT-qPCR) was used to detect the expression levels of target genes using the SYBR Green Master Mix (Applied Biosystems, CA) by recording Ct values. 18 S rRNA gene was utilized as the internal reference. After detecting the amplification efficiency’s consistency, the target genes’ relative expression levels in each sample were analyzed using the obtained Ct value versus the Ct value of 18 S rRNA. The relative expression of mRNA was calculated as 2^-ΔCt^×100%, ΔCt = Ct (target gene) -Ct(18 S rRNA). The primers’ list is reported in Supplementary Table 2.

### RNA sequencing analysis

RNA was extracted from KKU-156 and TBX3-overexpressing KKU-156 cells using the Zymo RNA Miniprep kit. The quality of RNA samples was determined by a Bioanalyzer (Agilent, CA). RNAs with RNA integrity number (RIN) values greater than 6.8 were eligible for RNA sequencing. Library preparation and sequencing were performed by Novogene (Novogene, CA). Read quality control was performed using fastqc (v0.11.7), and controlled reads were aligned to their respective genomes (Human Reference Genome Version 38 [hg38]). Bioconductor package org.Hs.eg.db (version 3.8.2) was used to convert Ensembl IDs of genes to Entrez IDs. Fragments per kilobase of transcript per million mapped reads (FPKM) after the deletion of duplicate genes were calculated and analyzed by the Edge R package (version 3.28.1). Relative RNA levels of each gene in both groups were estimated by the FPKM numbers.

### Transcription factor binding site prediction

The gene promoter sequence (first 2000 nucleotides in length) of TBX3 was obtained from the University of California, Santa Cruz (UCSC) Genome Browser (http://genome.ucsc.edu/). The transcription factor binding site prediction of selected oncogenes with TBX3 was determined through the JASPAR 2022 CORE database of transcription factor binding profiles [26], UCSC Genome Browser track data hub linked to JASPAR [27], and GeneCards.

### Chromatin immunoprecipitation (ChIP) assay

The ChIP assay was performed on KKU-156 and TBX3-overexpressing KKU-156 cells using the Zymo-Spin ChIP Kit following the manufacturer’s instructions (Cat #D5209, Zymo Research). In brief, after crosslinking the DNA and protein in cell samples using 1% formaldehyde, the crosslinked chromatin was sonicated (30 s “ON”, 30 s “OFF”, 10 cycles) at 4 °C using a Bioruptor (Diagenode, Denville, NJ) and sheared to approximately 100-500 bp. Immunoprecipitation was then performed with specific antibodies (exogenous TBX3: rabbit anti-HA tag, CST, #3724, 1:1000; endogenous TBX3: anti-TBX3 recombinant rabbit monoclonal antibody, Invitrogen, #702055, 1:250) at 4°C overnight. Rabbit anti-IgG (CST, #2729, 1:1000) was used as a negative control. DNA-protein complexes were pulled down by protein A magnetic beads and reverse-crosslinked to release DNA. Purified DNA was then used as a template for PCR. The input samples were the positive controls. PCR cycling conditions consisted of 5 min at 94 °C, followed by a three-step PCR program of 20 s at 94 °C, 30 s at 52 °C, and 30 s at 72 °C for a total of 35 cycles. Then, PCR products were run in a DNA gel to visualize the target bands. The primers specific for *MAD2L1* promoter region are *MAD2L1*-ChIP forward: 5′-GGGAGATCTGACTGAGTCTT-3′; *MAD2L1*-ChIP reverse: 5′- GTTGTTGGACTGAGGTCCCT-3′.

### Mouse treatment, tail vein hydrodynamic gene delivery, and lesions characterization

All animal studies were approved by the UCSF or University of Hawaii Cancer Center Animal Care and Use Committee (IACUC) and performed in specific pathogen-free facilities according to the NIH Principles of Laboratory Animal Care rules. Wild-type (WT) FVB/N mice were purchased from Charles River Laboratories (Wilmington, MA). *Tbx3*^*flox/flox*^ mice were kindly provided by Dr. Anne M. Moon (Weis Center for Research, Geisinger Clinic, PA). Hydrodynamic tail vein injections (HTVi) were performed on 4.5- to 6-week-old mice, as described previously [28, 29]. Briefly, for the *Akt/FBXW7ΔF/pT3* or *Akt/FBXW7ΔF/TBX3* model, 10 μg of *pT3-EF1α-HA-myr-Akt* (human), 40 μg of *pT3-EF1α-HA-Fbxw7ΔF* (human) and 30 μg of *pT3* (vector), or 30 μg of *pT3-EF1α-HA-TBX3* (human) were coinjected into each FVB/N mouse along with 3.2 μg of pCMV-Sleeping Beauty transposase in 2 mL of sterile saline. For the *Akt/FBXW7ΔF/pCMV* or *Akt/FBXW7ΔF/Cre* model, 10 μg of *pT3-EF1α-HA-myr-Akt* (human), 40 μg of *pT3-EF1α-HA-Fbxw7ΔF* (human) and 60 μg of *pCMV*, or 60 μg of *pCMV-Cre* were coinjected into each *Tbx3*^*flox/flox*^ mice along with 4.4 μg of pCMV-Sleeping Beauty transposase in 2 mL of sterile saline. For the *Akt/NICD/sgEGFP* or *Akt/NICD/sgMAD2L1* model, 20 μg of *pT3-EF1α-HA-myr-Akt* (human), 20 μg of *pT3-EF1α-NICD* (human) and 40 μg of *pX330-sgeGFP* or 40 μg of *pX330-sgMAD2L1* were coinjected into each FVB/N mouse along with 1.6 μg of pCMV-Sleeping Beauty transposase in 2 mL of sterile saline. For the *Akt/FBXW7ΔF/sgEGFP* or *Akt/FBXW7ΔF/sgMAD2L1* model, 10 μg of *pT3-EF1α-HA-myr-Akt* (human), 40 μg of *pT3-EF1α-HA-Fbxw7ΔF* (human) and 40 μg of *pX330-sgGFP*, or 40 μg of *pX330-sgMAD2L1* were coinjected into each FVB/N mouse along with 2 μg of pCMV-Sleeping Beauty transposase in 2 mL of sterile saline. Mice were euthanized when a high tumor burden occurred (liver weight > 4.5 g). A certified pathologist and liver expert (M.E.) analyzed all mouse and human lesions.

### Human tissue samples

Human iCCA and corresponding surrounding non-tumorous liver tissues (*n* = 82 for each group) were collected at the University of Regensburg (Regensburg, Germany). The local Ethical Committee of the Medical University of Regensburg (approval code: 17-1015-101) provided the Institutional Review Board approval in compliance with the Helsinki Declaration. Written informed consent was obtained from all individuals.

### Hematoxylin-eosin (H&E), immunohistochemistry (IHC), and proliferation index

Dissected mouse liver tissues were fixed in 10% neutral buffered formalin (Thermo Fisher Scientific, Waltham, MA) at room temperature for one to three days. Next, the tissues were transferred to 70% ethanol and soaked until paraffin embedding. Paraffin-embedded tissues were cut into 5 µm thick sections for H&E or IHC staining. For H&E staining, tissue sections were subjected to standard procedures, including deparaffining, hematoxylin and eosin staining, dehydration, and mounting. For IHC staining of mouse lesions, tissue sections were preheated in an oven at 60 °C overnight. Next, the slides were placed in 10 mM sodium citrate buffer (pH 6.0) for antigen retrieval after deparaffinization. Subsequently, tissue sections were blocked in 10% goat serum and Avidin-Biotin blocking reagent (Vector Laboratories, Burlingame, CA), followed by incubation with primary antibodies overnight at 4 °C. Primary antibodies include rabbit anti-Ki67 (1:150, Thermo Fisher Scientific, #RM-9106-S1), HNF4α (1: 2000, Abcam, #Ab181604), CK19 (1:1000, Abcam, #Ab133496), Vimentin (1:50, Cell Signaling Technology, #5741), Sox9 (1:2000, Abcam, #Ab185230), and YAP (1:100, Cell Signaling Technology, #10474). The next day, tissue sections were treated with 3% H_2_O_2_ to quench endogenous peroxidase activity and incubated with goat anti-rabbit secondary antibody (1:500, Invitrogen, #B2770) for 30 min at room temperature. Finally, tissue sections were incubated with the Vectastain Elite ABC kit (Vector Laboratories) and DAB substrate (Dako North America, Carpinteria, CA) for signal visualization. Subsequently, the slides were counterstained with hematoxylin and mounted after dehydration (StatLab, Lodi, CA). When the mounted slides were dehydrated, images of the slides were taken under a Leica inverted microscope. Seven to 8 field views were selected for each tissue section. Human specimens were harvested and fixed in 10% formalin overnight at 4°C and embedded in paraffin. Hematoxylin and eosin (Thermo Fisher Scientific, Waltham, MA, USA) staining was conducted using a standard protocol. For immunohistochemistry, antigen retrieval was achieved by boiling the slides in 10 mM sodium citrate buffer (pH 6.0) and then cooling down at room temperature for 30 min. For TBX3 staining, antigen unmasking was achieved using the SignalStain^®^ EDTA Unmasking Solution from Cell Signaling Technology, Danvers, MA), following the manufacturer’s instructions. After blocking with 5% goat serum and the Avidin-Biotin blocking kit (Vector Laboratories, Burlingame, CA, USA), the slides were incubated with the anti-p-AKT (Ser473; dilution 1:100; #4060; Cell Signaling Technology), anti-FBXW7 (dilution 1:100; #28424-1-AP; Proteintech, Rosemont, IL), anti-TBX3 (dilution 1:50; # 16741-1-AP; Proteintech), anti-HNF4α (dilution 1:100; #ab181604; Abcam, Waltham, MA), anti-CK19 (dilution 1:400; #ab133496; Abcam), anti-SATB2 (dilution 1:300; #ab227686; Abcam), anti-RANKL (1:500; #23408-1-AP; Proteintech), and Ki-67 (dilution 1:800; #9449; Cell Signaling Technology) primary antibodies overnight at 4 °C. Subsequently, slides were subjected to 3% hydrogen peroxide for 10 min to quench endogenous peroxidase activity, and the biotin-conjugated secondary antibody was applied at a 1:500 dilution for 30 min at room temperature. The reaction was visualized with the Vectastain ABC-Elite Peroxidase Kit (Vector Laboratories, #PK-6100) with ImmPACT DAB (Vector Laboratories, SK-4105) as the chromogen. Finally, slides were counterstained with Mayer’s hematoxylin. Tumor proliferation index was determined in human iCCA lesions livers by counting Ki67 positive cells on at least 2000 tumor cells per sample.

### Statistical analysis

In vitro, mouse, and human data were analyzed and processed using the GraphPad Prism 9.0 software (GraphPad, San Diego, CA). Both in vitro and in vivo mouse data were analyzed by a 2-tailed unpaired Student’s *t* test. Mice survival curve was generated using the Kaplan–Meier method and subsequently compared using the log-rank test. For human data, comparisons between the two groups were performed using the Mann–Whitney *U* test for non-parametric data or the unpaired *t*-test for parametric data. Survival curves were analyzed using the Log-rank (Mantel-Cox) test. Data are presented as mean ± SD. *p* < 0.05 was considered statistically significant.

## Results

### Overexpression of TBX3 inhibits iCCA cell growth in vitro

**Fig. 1:**
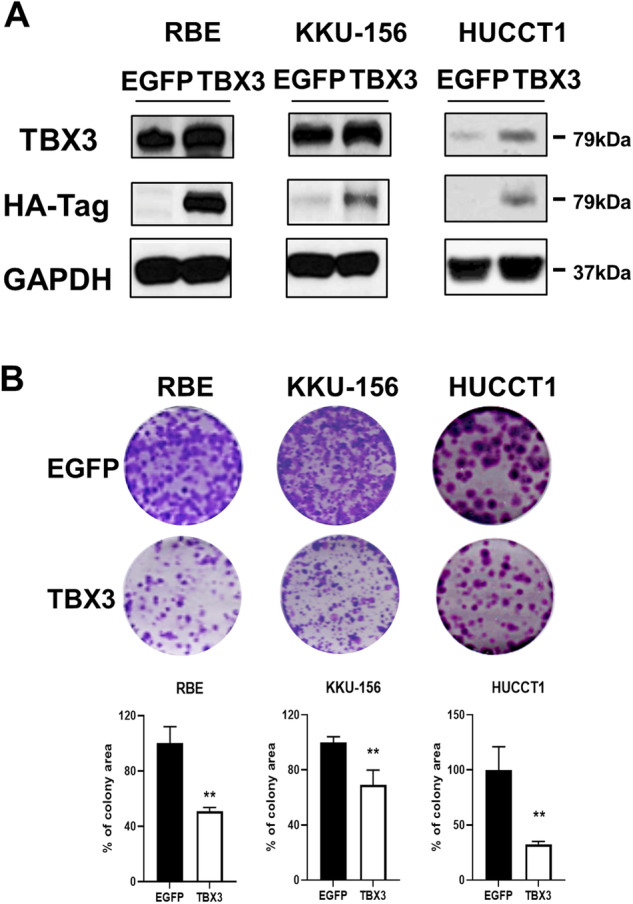
Overexpression of TBX3 impedes the growth of human intrahepatic cholangiocarcinoma in vitro. **A** Western blot analysis determined the levels of TBX3 and HA-Tag proteins in RBE, KKU-156, and HUCCT1 cells. GAPDH serves as a loading control. **B** Representative images of colonies in RBE, KKU-156, and HUCCT1 cells transfected with EGFP or TBX3 and acquired one week after incubation. The bar graphs illustrate the colony area percentage of each group quantified using the ImageJ software. **: Student’s *t* test, *p* < 0.01.

First, to investigate the functional role of TBX3 in iCCA, we overexpressed HA-tagged TBX3 in human iCCA cell lines, including RBE, KKU-156, and HUCCT1 cells. RBE and KKU-156 cells display elevated protein levels of TBX3, whereas TBX3 was low in HUCCT1 cells (Fig. 1A). Western blot analysis confirmed the successful overexpression of TBX3 protein in the three iCCA cell lines used (Fig. 1A). Importantly, colony formation assays demonstrated that the overexpression of TBX3 strongly inhibited iCCA cell growth in vitro compared with controls (Fig. 1B), suggesting that overexpression of TBX3 inhibits iCCA growth.

### Overexpression of TBX3 delays mouse cholangiocarcinogenesis in vivo

**Fig. 2:**
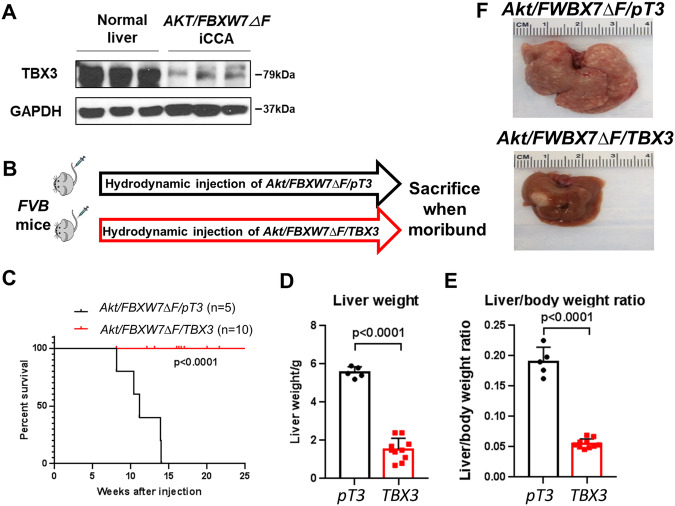
Overexpression of TBX3 inhibits mouse iCCA growth. **A** Protein levels of TBX3 in normal livers and *Akt/FBXW7ΔF* iCCA tumors. GAPDH was used as the loading control. **B** Study design. FVB mice were hydrodynamically injected with *Akt/FBXW7ΔF/pT3* or *Akt/FBXW7ΔF/TBX3* plasmids. Mice were sacrificed when bearing large tumors (tumor weight > 4.5 g). **C** The mice survival curve was plotted when reaching the endpoint. **D**, **E** Endpoint liver weight (**D**) was recorded, and liver weight to body weight ratios (**E**) were calculated. Student’s *t* test: *p* < 0.0001. **F** Representative gross liver from *Akt/FBXW7ΔF/pT3* or *Akt/FBXW7ΔF/TBX3* mice at the respective endpoint.

Subsequently, we investigated whether Tbx3 could affect iCCA formation in vivo. For this purpose, we applied a previously established murine iCCA model, induced by the co-expression of an activated form of *Akt* (*myr-Akt*) and the dominant negative form of *FBXW7* (*FBXW7ΔF*) (*Akt/FBXW7ΔF*) in the mouse liver via hydrodynamic tail vein gene injection (HTVi) [30]. In this model, iCCA lesions develop by ~7 weeks post-injection [30]. This model was chosen as we have demonstrated that it is highly relevant to human cholangiocarcinogesis [30]. In addition, Tbx3 protein levels were low in the lesions from this model compared to normal livers of uninjected mice (Fig. 2A), supporting it as an ideal model to investigate the effects of Tbx3 overexpression in iCCA.

Thus, we co-overexpressed *TBX3* and *Akt/FBXW7ΔF* plasmids in the mouse liver via HTVi (herein referred to as *Akt/FBXW7ΔF/TBX3* mice). As the control group, additional mice were co-injected with the *pT3EF1α* empty vector (*Akt/FBXW7ΔF/pT3)* (Fig. 2B). Mice were monitored for tumor growth and euthanized if they showed a sizeable abdominal mass, became moribund, or until ~25 weeks post-injection (end of the experiment). All *Akt/FBXW7ΔF/pT3* injected mice developed a considerable liver tumor burden and required euthanasia between 8 and 14 weeks post-injection. In striking contrast, none of the *Akt/FBXW7ΔF/TBX3* injected mice showed any sign of abdominal mass. All mice appeared healthy when harvested around 25 weeks post-injection (Fig. 2C). The liver weight and liver/body ratio were significantly lower in *Akt/FBXW7ΔF/TBX3* mice than in control *Akt/FBXW7ΔF/pT3* mice (Fig. 2D–F).

**Fig. 3:**
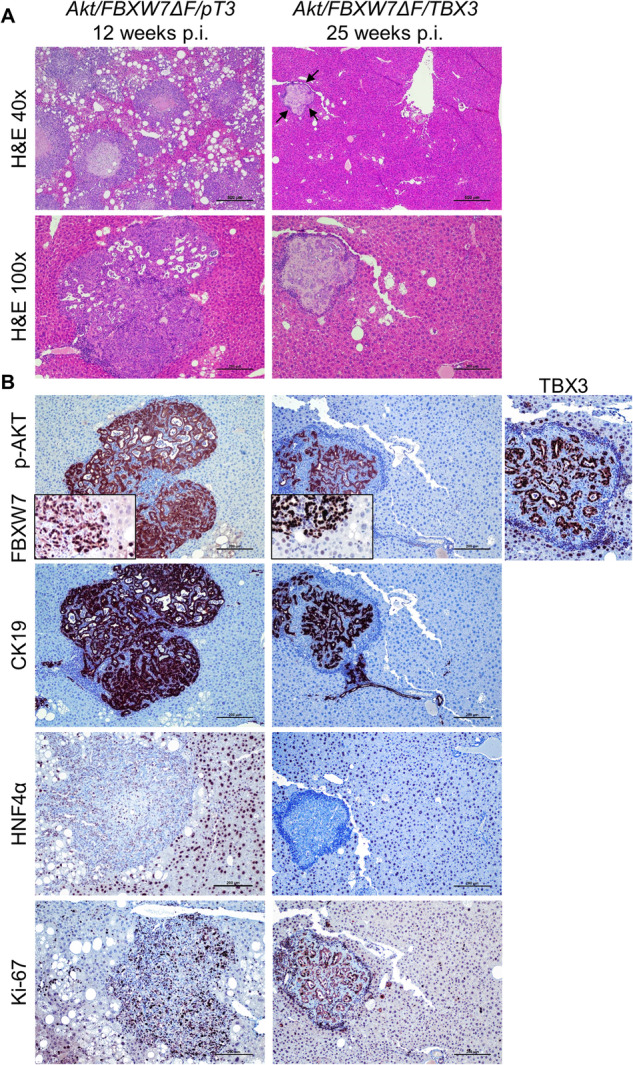
Overexpression of TBX3 delays *Akt/FBXW7ΔF-*driven cholangiocarcinogenesis. **A** H&E staining of liver tissues from *Akt/FBXW7ΔF/pT3* (left column) or *Akt/FBXW7ΔF/TBX3* (right column) mice harvested at the specific time points as indicated. Arrows: tumor nodules observed in the mouse liver tissues. **B** Immunohistochemical staining of liver tissues from *Akt/FBXW7ΔF/pT3* (left column) or *Akt/FBXW7ΔF/TBX3* (right column) mice. Original magnifications: ×40 in the upper panel of (**A**); ×200 in insets; ×100 in all the other panels; scale bar: 500 µm in (**A**); 100 µm in all the other panels. H&E, hematoxylin and eosin staining.

H&E staining revealed that tumor lesions occupied most of the *Akt/FBXW7ΔF/pT3* liver tissues harvested at 12 weeks post-injection (Fig. 3A). In contrast, in *Akt/FBXW7ΔF/TBX3* mice harvested at 25 weeks post-injection, only a few sporadic small iCCA lesions could be identified by H&E staining (Fig. 3A). As expected, all tumor iCCA lesions in both cohorts were positive for p-AKT and the biliary epithelial marker CK19 and negative for the HNF4α hepatocyte marker (Fig. 3B). The overexpression of TBX3 was validated by the strong TBX3 IHC signal in *Akt/FBXW7ΔF/TBX3* liver tumor tissues (Fig. 3B). Furthermore, Ki67 immunostaining showed significantly lower proliferating cells in *Akt/FBXW7ΔF/TBX3* lesions (Fig. 3B), providing a possible mechanism by which TBX3 regulates iCCA progression.

Notably, in addition to the iCCA lesions, 3 of 10 *Akt/FBXW7ΔF/TBX3* mouse livers showed the presence of mesenchymal lesions (Supplementary Fig. 1). Histologically, these lesions were characterized by spindle/sarcomatoid cells immersed in an abundant, densely-packed matrix. Furthermore, these lesions displayed the focal deposition of an osteoid substance, suggesting the presence of osteosarcoma-like lesions. In addition, the osteoid substance was demarcated by multinucleated cells resembling osteoclasts. The positive immunoreactivity of the mesenchymal cells for the SATB2 transcription factor and the RANK ligand (RANKL) further substantiated the presumable osteoid nature of these lesions. Also, these lesions exhibited intense and concomitant immunoreactivity for Akt, FBXW7, and TBX3 proteins, implying their origin from the injected genes, and were negative for the CK19 cholangiocellular marker. Moreover, they showed elevated immunopositivity for the proliferation marker Ki67, suggesting their malignant nature.

In summary, the data demonstrate that overexpression of *TBX3* strongly inhibits *Akt/FBXW7ΔF* driven iCCA formation in mice. Interestingly, TBX3 overexpression can cause the development of mesenchymal tumors with osteoid differentiation in a subset of these mice.

### Ablation of *Tbx3* accelerates *Akt/FBXW7ΔF* driven cholangiocarcinogenesis

**Fig. 4:**
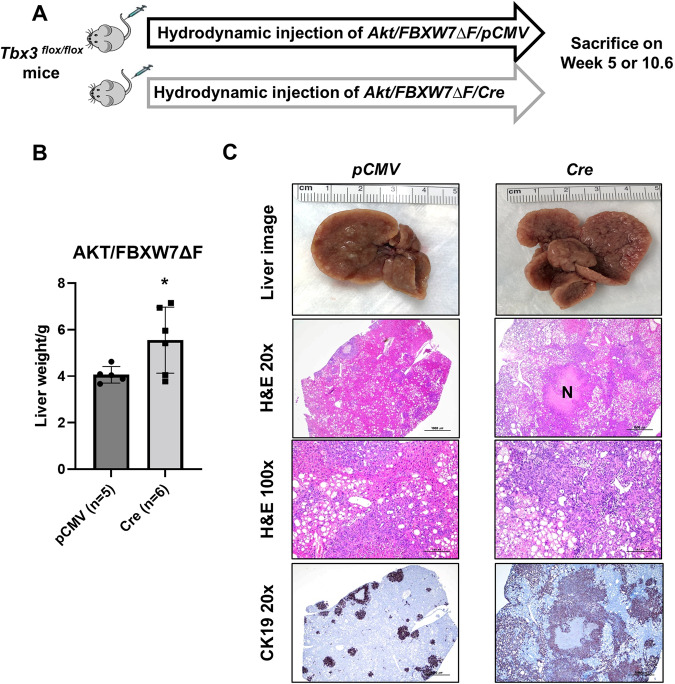
Knocking down *Tbx3* accelerates iCCA tumor development in the *Akt/FBXW7ΔF* model. **A** Study design. *Tbx3*^*flox/flox*^ conditional knockout mice were hydrodynamically injected with *Akt/FBXW7ΔF/pCMV* or *Akt/FBXW7ΔF/Cre* plasmids. Mice were sacrificed when they were moribund. **B** The liver weight of mice in each group. *p < 0.05. **C** Representative gross liver images, H&E stainings, and IHC images for CK19 of *Akt/FBXW7ΔF/pT3* (left column) or *Akt/FBXW7ΔF/Cre* (right column). Large areas of necrosis (N) could be detected in these lesions. Original magnifications: ×20 in H&E and CK19 ×20; ×100 in H&E ×100; scale bar: 1000 µm in H&E and CK19 ×20; 100 µm in H&E ×100.

Next, we asked whether deletion of *Tbx3* would accelerate iCCA tumorigenesis in vivo. For this purpose, we used conditional *Tbx3* knockout (*Tbx3*^*flox/flox*^) mice [21]. Thus, we injected *Akt, FBXW7ΔF*, and *pCMV/Cre* plasmids into *Tbx3*^*flox/flox*^ mice (*Akt/FBXW7ΔF/Cre*). This strategy allows the expression of *Akt/FBXW7ΔF* into *Tbx3*-null liver cells. Additional *Tbx3*^*flox/flox*^ mice were injected with the *Akt/FBXW7ΔF/pCMV* (with pCMV empty vector) plasmids as control (Fig. 4A). All mice were harvested around 11 weeks post-injection. *Akt/FBXW7ΔF/Cre* injected mice demonstrated significantly increased liver weight compared to the *Akt/FBXW7ΔF/pCMV* injected mice (Fig. 4B). The histopathological analysis also confirmed the increased number and size of iCCA lesions in *Akt/FBXW7ΔF/Cre* mice (Fig. 4C). Although more and larger iCCA lesions developed in the *Akt/FBXW7ΔF/Cre* mouse liver, no histomorphologic differences were detected between *Akt/FBXW7ΔF/pCMV* and *Akt/FBXW7ΔF/Cre* tumors. Indeed, tumors consisted of iCCA lesions admixed with foci of lipid-rich hepatocytes in both mouse groups (Fig. 4C).

In summary, ablation of *Tbx3* accelerates *Akt/FBXW7ΔF*-driven cholangiocarcinogenesis. The effects were relatively moderate, likely due to the already low levels of *Tbx3* in *Akt/FBXW7ΔF* mouse iCCA lesions (Fig. 2A).

### MAD2L1 is a TBX3 target gene in iCCA

**Fig. 5:**
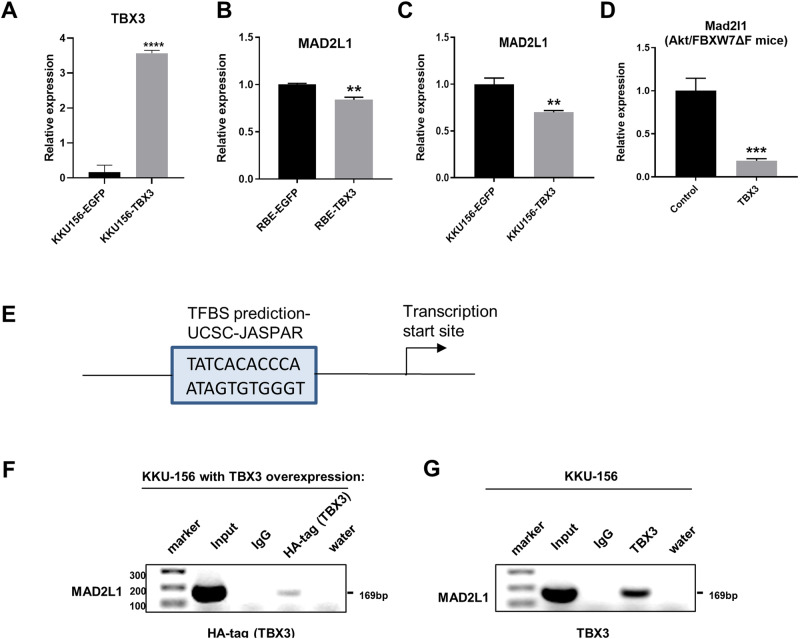
MAD2L1 is negatively regulated by TBX3 and serves as a target of TBX3. **A** Validation of mRNA-level upregulation of *TBX3* in TBX3-overexpressing KKU-156 cells. **B**–**D** The expression levels of *MAD2L1* were measured using qPCR in parental and TBX3-overexpressing iCCA cell lines and mouse livers. **B** Parental and TBX3-overexpressing RBE cells. **C** Parental and TBX3-overexpressing KKU-156 cells. **D** Liver tissues from *Akt/FBXW7ΔF/pT3* and *Akt/FBXW7ΔF/TBX3* mice. **E** The putative binding site of TBX3 on *MAD2L1* promoter region. **F**, **G** ChIP of MAD2L1 tested by PCR and DNA gels. Left panel (**F**) used TBX3-overexpressing KKU-156 cells and exogenous HA tag antibody. Right panel (**G**) KKU-156 cells and endogenous TBX3 antibody.

Our present data indicate that TBX3 overexpression suppresses cholangiocarcinogenesis. Therefore, we sought to identify TBX3 targets whose inactivation hinders tumorigenesis. Previous studies have shown that TBX3 is a transcriptional repressor [16]. To identify the genes regulated by TBX3, we overexpressed TBX3 in the KKU156 human iCCA cell line and performed RNA-Seq experiments. The analysis revealed 827 significantly upregulated and 904 downregulated genes following TBX3 overexpression (Supplementary Table 3 and Supplementary Fig. 2A). *TBX3* was identified as a top-upregulated gene, confirming its successful overexpression in the transfected cells (Supplementary Table 3 and Fig. 5A)

Since TBX3 is a well-characterized transcriptional repressor, we focused on the genes downregulated by TBX3 overexpression. Pathway analysis revealed that “Ribosome”, which is required for protein translation, was the most downregulated pathway following TBX3 overexpression (Supplementary Fig. 2B). Furthermore, cell metabolic cascades, such as oxidative phosphorylation, purine, arginine, and proline metabolism were downregulated in KKU-156 TBX3 cells (Supplementary Fig. 2B). In addition, several genes involved in cell proliferation, such as *CDK1*, *CCNG1*, and *PLK4*, and in epithelial-mesenchymal transition (EMT) such as *ZEB2*, were downregulated by TBX3 (Supplementary Table 3), suggesting that TBX3 might suppress iCCA growth via multiple mechanisms.

Next, we searched for a TBX3 target gene that might have a crucial function in iCCA. Among the downregulated genes, we first narrowed down the candidate genes overexpressed in iCCA samples based on the TCGA-CHOL and NCI-CCA databases [24, 25]. Subsequently, we calculated the correlation between *TBX3* expression and that of the iCCA-overexpressed genes in the NCI-CCA database, as this dataset has a larger sample size than the TCGA-CHOL study. We selected the top 30 candidate genes from the analyses. Finally, we predicted whether TBX3 could bind to the promoter region of these genes. Among the 30 top candidate genes, eight genes displayed the predicted TBX3 binding site on their promoter sequences. These genes were selected for additional studies (Supplementary Table 4).

Afterward, we performed q-RT-PCR validation of the eight candidate genes in iCCA cells and mouse tumor samples. Specifically, we determined the expression of these transcripts in the RBE and KKU-156 cell lines, two human iCCA cell lines with detectable TBX3 expression at baseline (Supplementary Fig. 3A, B), with or without TBX3 overexpression. In addition, we assessed the levels of these transcripts in iCCA lesions from *Akt/FBXW7ΔF/pT3* and *Akt/FBXW7ΔF/TBX3* mice (Supplementary Fig. 3C). The mitotic arrest deficient 2 like 1 (*MAD2L1*) gene was the only one consistently downregulated by TBX3 in the KKU156 and REB cells and mouse *Akt/FBXW7ΔF* iCCA lesions (Fig. 5B–D and Supplementary Fig. 3), suggesting that *MAD2L1* expression is regulated by TBX3 in iCCA.

Next, we determine whether MAD2L1 is a direct target of TBX3 in iCCA cells. A candidate TBX3 binding motif was identified at the TBX3 promoter region (Fig. 5E). We performed chromatin immunoprecipitation (ChIP) analysis using KKU-156 cells with overexpression of TBX3 (with HA-tag). ChIP-PCR analysis using an anti-HA tag antibody confirmed that the overexpressed HA-tagged TBX3 could bind to the *MAD2L1* promoter sequence (Fig. 5F). Next, we performed ChIP-PCR using an anti-TBX3 antibody and untransfected KKU-156 cells. Again, the ChIP-PCR experiment confirmed the binding of endogenous TBX3 to the *MAD2L1* promoter region (Fig. 5G).

The data indicate that TBX3 directly binds to the MAD2L1 promoter and represses its expression in iCCA cells.

### MAD2L1 promotes iCCA growth in vitro

To delineate the MAD2L1 function in iCCA, we overexpressed the *MAD2L1* gene in iCCA cells. qRT-PCR confirmed the successful upregulation of *MAD2L1*. Subsequent colony formation assay showed that overexpression of MAD2L1 could accelerate human iCCA cell growth (Supplementary Fig. 4). Then, we deleted *MAD2L1* in RBE and HUCCT1 cells using CRISPR-Cas9-based gene editing. Knocking out *MAD2L1* effectively inhibited the proliferation of both RBE and HUCCT1 cells (Supplementary Fig. 5A). Western blot analysis and Sanger sequencing confirmed the efficient deletion of the *MAD2L1* gene in the iCCA cells (Supplementary Fig. 5B, C). These data indicate that MAD2L1 is a relevant regulator of iCCA growth in vitro.

### Deletion of *Mad2l1* prevents *Akt/FBXW7ΔF*-driven iCCA development in mice

**Fig. 6:**
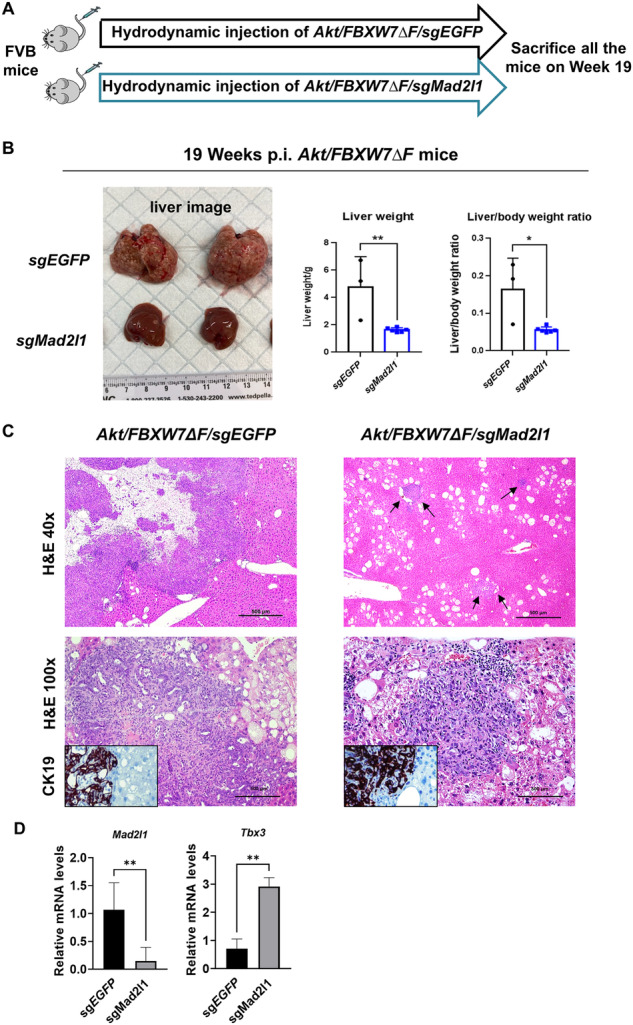
Deletion of *Mad2l1* hampers *Akt/FBXW7ΔF*-driven cholangiocarcinogenesis. **A** Study design. FVB mice were hydrodynamically injected with *Akt/FBXW7ΔF/sgEGFP* or *Akt/FBXW7ΔF/sgMad2l1* plasmids. Mice were sacrificed on week 19 post-injection. **B** Representative gross liver images and the liver weight of mice in each group. **C** H&E and IHC of representative livers from each group. Arrows indicate tumor foci. Original magnifications: ×40 in the upper panels; ×100 in the lower panels; ×200 in insets. Scale bar: 500 µm in the upper panels; 200 µm in the lower panels. Student’s *t* test, *p < 0.05; **p < 0.01. **D** qRT-PCR analysis of *Tbx3* and *Mad2l1* mRNA expression in *Akt/FBXW7ΔF/sgEGFP* or *Akt/FBXW7ΔF/sgMad2l1* liver tissues.

Our previous findings showed that overexpression of *TBX3* inhibited *Akt/FBXW7ΔF*-dependent iCCA formation in mice. In addition, overexpression of *TBX3* suppressed *MAD2L1* expression. Based on these data, we hypothesized that deleting *Mad2l1* could also hamper *Akt/FBXW7ΔF*-driven-cholangiocarcinogenesis in mice. To test this hypothesis, we designed a sgRNA against mouse *Mad2l1* and cloned it into pX330 plasmid. Next, we co-injected *Akt/FBXW7ΔF* vectors with *pX330-sgMad2l1* (*Akt/FBXW7ΔF/sgMad2l1*). This strategy allowed the expression of *Akt/FBXW7ΔF* oncogenes in *Mad2l1-*deleted mouse liver cells. Additional mice were injected with *Akt/FBXW7ΔF* plasmids with *pX330-sgEGFP* (*Akt/FBXW7ΔF/sgEGFP*) as the control (Fig. 6A). Upon euthanasia, all *Akt/FBXW7ΔF/sgEGFP* mice demonstrated high liver tumor burden. In contrast, *Akt/FBXW7ΔF/sgMad2l1* mice showed few liver tumor nodules (Fig. 6B). Histologically, liver lesions developed in *Akt/FBXW7ΔF/sgEGFP* and *Akt/FBXW7ΔF/sgMad2l1* mice were identical, consisting of iCCA surrounded by lipid-rich hepatocytes (Fig. 6C). The effectiveness of *sgMad2l1* mediated gene editing was validated via PCR analysis of genomic DNA from *Akt/FBXW7ΔF/sgMad2l1* mouse liver tissues (Supplementary Fig. 6). Furthermore, qRT-PCR analysis confirmed lower expression of *Mad2l1* and higher expression of *Tbx3* in *Akt/FBXW7ΔF/sgMad2l1* liver tissues than in *Akt/FBXW7ΔF/sgEGFP* liver tissues (Fig. 6D).

In summary, knockout of *Mad2l1* strongly inhibits *Akt/FBXW7ΔF*-dependent cholangiocarcinogenesis.

### Deletion of *Mad2l1* hampers *Akt/NICD*-driven iCCA development in mice

To confirm our preliminary findings about *MAD2L1* and rule out the possibility that its suppression of iCCA development only applies to the *Akt/FBXW7ΔF* model, we employed the *Akt/NICD* mouse iCCA model and silenced *Mad2l1*. Specifically, we hydrodynamically injected *Akt/NICD/sgEGFP* or *Akt/NICD/sgMad2l1* plasmids in FVB/N mice (Supplementary Fig. 7A). At week 5 post-injection, mice were sacrificed, and the liver weight was recorded. Downregulation of *Mad2l1* significantly reduced the liver weight and liver versus body weight ratio of *Akt/NICD* mice (Supplementary Fig. 7B). Histologically, the *Akt/NICD/sgMad2l1* group displayed sparse or no tumor nodules on the liver surface. In contrast, the *Akt/NICD/sgEGFP* group exhibited many tumor nodules, thereby confirming that knockout of *Mad2l1* can suppress iCCA growth and development in the *Akt/NICD* tumor model (Supplementary Fig. 7B). Additionally, qRT-PCR analysis confirmed that lower expression of *Mad2l1* and higher expression of *Tbx3* in *Akt/NICD/sgMad2l1* liver tissues than those in *Akt/NICD/sgEGFP* liver tissues (Supplementary Fig. 7C).

The data show that MAD2L1 is upregulated, and TBX3 represses its expression. Overexpression of TBX3 or deletion of MAD2L1 leads to iCCA growth inhibition, suggesting that the TBX3/MAD2L1 pathway regulates iCCA pathogenesis.

### TBX3 and MAD2L1 in human iCCA samples

**Fig. 7:**
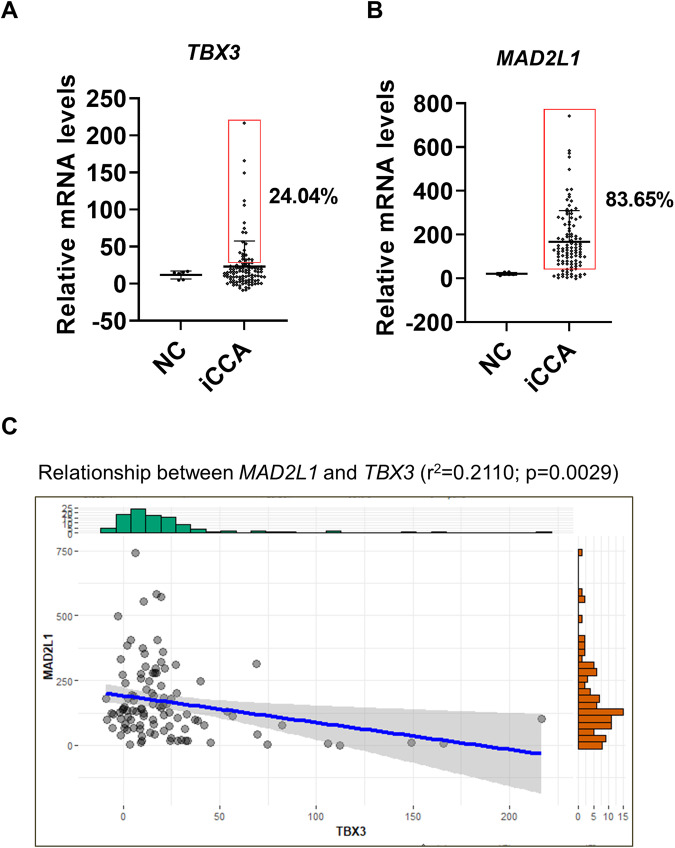
*TBX3* and *MAD2L1* mRNA expression in human iCCA samples. **A** *TBX3* mRNA expression in human iCCA samples in comparison with normal cholangiocytes (NC) based on the NCI dataset. The samples whose expression levels were higher than Average(NC) + 3*StandardDeviationn(NC) were considered to be higher expressed and represented in the box area. **B** *MAD2L1* mRNA expression in human iCCA samples in comparison with normal cholangiocytes (NC) based on NCI dataset. The samples whose expression levels were higher than Average(NC) + 3*StandardDeviationn(NC) were considered to be higher expressed and represented in the box area. *p < 0.05. **C** The relationship between *TBX3* and *MAD2L1* mRNA levels in the NCI database.

We investigated the expression of TBX3 and MAD2L1 in human iCCA samples. First, we analyzed *TBX3* and *MAD2L1* levels in the NCI iCCA dataset because it contains the expression of micro-dissected normal cholangiocytes from the liver (*n* = 6), which we used as the normal control for iCCA [24]. We found that *TBX3* expression was upregulated in ~24%, and *MAD2L1* expression is upregulated in ~84% of human iCCA samples, respectively (Fig. 7A, B). Importantly, the *TBX3* and *MAD2L1* expression demonstrated a strong negative correlation (Fig. 7C), consistent with our studies that *TBX3* negatively regulates the expression of *MAD2L1*.

To further analyze the possible functional roles of *TBX3* in iCCA, we identified genes whose expression levels are positively or negatively correlated with *TBX3* mRNA level. Due to the larger sample size of NCI iCCA studies (*n* = 104), we used this dataset for these analyses. We identified 4761 *TBX3* positively correlated genes and 2475 *TBX3* negatively correlated genes. The pathway analysis revealed that multiple cancer-related gene lists, such as “Renal cell carcinoma”, “Hepatocellular carcinoma”, and “Cell cycle”, were enriched in *TBX3* negatively correlated genes (Supplementary Fig. 8). For example, the expression of *CCNE1, CCNB1, PLK1, CDK4*, and *PCNA* was found to be negatively correlated with *TBX3* mRNA expression (Supplementary Fig. 9). The data are consistent with our data showing the overexpression of TBX3 inhibits iCCA growth.

**Fig. 8:**
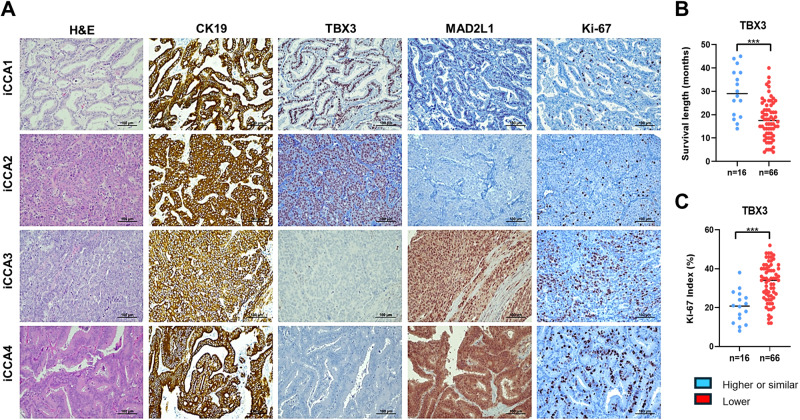
Representative immunohistochemical patterns of TBX3 and MAD2L1 proteins in human intrahepatic cholangiocarcinoma specimens (iCCA; *n* = 82). **A** The top panels show an example of human iCCA (denominated iCCA1) displaying high nuclear levels of TBX3, faint expression of MAD2L1, and low proliferative activity (the latter indicated by Ki-67 staining). A second tumor (iCCA2) showed intense and homogeneous immunoreactivity for TBX3, absent MAD2L1 immunolabeling, and low proliferation. A third (iCCA3) and fourth (iCCA4) tumor lacked TBX3 immunoreactivity and exhibited pronounced cytoplasmic and nuclear immunoreactivity for MAD2L1, which are paralleled by elevated proliferation. Cytokeratin 19 (CK19) was used as a biliary marker. Abbreviation: H&E, hematoxylin and eosin staining. Original magnification: 200× in all the panels. Scale bar: 100 µm in all the panels. **B** iCCA specimens showing TBX3 immunoreactivity (*n* = 16) displayed significantly longer survival than TBX3-negative tumors. **C** iCCA specimens showing TBX3 immunoreactivity (*n* = 16) displayed significantly lower proliferative activity than TBX3-negative tumors, as assessed by Ki-67 index (% positive cells/2000 cells). Student’s *t* test: ***p < 0.0001.

Next, we analyzed the protein expression of TBX3 and MAD2L1 in a collection of human iCCA samples for which the survival data were available (*n* = 82; Table 1). By immunohistochemistry (Supplementary Fig. 10), we found that hepatocytes and cholangiocytes in the surrounding neoplastic tissues displayed moderate nuclear staining for TBX3. In addition, sixty-six iCCA (80.5%) were negative for TBX3 staining, whereas sixteen tumors (19.5%) displayed homogeneous nuclear immunoreactivity for the protein, which was higher or comparable to that in non-tumorous livers (Fig. 8). Of note, the tumor subset displaying high levels of TBX3 exhibited higher survival length and lower Ki-67 index compared with TBX3-negative tumors (Fig. 8 and Table 1). No other association between TBX3 expression and clinical/pathological features of iCCA was noted (Table 1). As concerns MAD2L1, immunoreactivity was moderate in the cytoplasm of hepatocytes and absent in cholangiocytes (Supplementary Fig. 10). In contrast, MAD2L1 was strongly expressed in the nucleus and cytoplasm of most iCCA samples (68/82, 82.3%), while no MAD2L1 immunolabeling could be observed in the remaining 14 tumor specimens (Fig. 8). Notably, 10 of 14 (71.4%) iCCA exhibiting low MA2DL1 staining revealed simultaneous upregulation of TBX3, further supporting MAD2L1 as a TBX3 target in iCCA.

**Table 1:** Clinicopathological features of iCCA patients.

Variable	TBX3 IHC(%)		Total	*P* value
	Negative	Positive		
Survival, (months)	17.5(11.8,24.0)	29.0(19.3,38.0)	19.0(14.0,26.0)	<0.001^a^
*Sex*				
F	25(37.88)	6(37.50)	31(37.80)	0.978^b^
M	41(62.12)	10(62.50)	51(62.20)	
*Cirrhosis*				0.259^b^
No	49(74.24)	14(87.50)	63(76.83)	
Yes	17(25.76)	2(12.50)	19(23.17)	
*Tumor size*				0.558^b^
<5 cm	49(74.24)	13(81.25)	62(75.61)	
>5 cm	17(25.76)	3(18.75)	20(24.39)	
*Lymph Node Metastasis*				0.416^b^
No	47(71.21)	13(81.25)	60(73.17)	
Yes	19(28.79)	3(18.75)	22(26.83)	
*Differentiation*				0.775^b^
Moderately	19(28.79)	4(25.00)	23(28.05)	
Poorly	15(22.73)	5(31.25)	20(24.39)	
Well	32(48.48)	7(43.75)	39(47.56)	
*Tumor number*				0.932^b^
Multiple	13(19.70)	3(18.75)	16(19.51)	
Single	53(80.30)	13(81.25)	66(80.49)	
*Etiology*				0.836^b^
Virus	27(40.91)	7(43.75)	34(41.46)	
Others	39(59.09)	9(56.25)	48(58.54)	

^a^*t* test. bChi-square test.

^b^Chi-square test.

In summary, our data demonstrate that TBX3 is overexpressed in a subset of human iCCA samples, and its high expression is associated with decreased MAD2L1 levels, decreased tumor cell proliferation, and prolonged survival.

## Discussion

In the current study, we established the role of the TBX3 transcription factor in iCCA using human cell lines, mouse models, and human samples. Overexpression of *TBX3* resulted in diminished iCCA cell growth in vitro and in vivo, and its genetic deletion caused an increase in iCCA aggressiveness in all models. Similarly, we previously showed that TBX3 expression was associated with less aggressive biological behavior and a more differentiated phenotype in a β-catenin-driven HCC model [21]. An opposite, pro-oncogenic role for TBX3 has been revealed in other tumor types, such as malignant melanoma, breast, gastric, and cervical cancers [1–3]. These contrasting data suggest that TBX3 possesses significant functional plasticity across cancers as a tumor suppressor or promoter. This diverse behavior may be due to the expression of alternatively spliced isoforms [31] and the cell/tissue context. In this regard, the interaction with tissue- or tumor-specific transcription factors might dictate the net effect of TBX3 in a particular tumor type.

Interestingly, besides demonstrating that overexpression of TBX3 inhibits iCCA growth, TBX3 overexpression induced mesenchymal lesions of osteoid nature in a subset of *Akt/FBXW7ΔF* mice. The reason why only some *Akt/FBXW7ΔF/TBX3* mice developed mesenchymal/ osteosarcoma-like tumors is unclear. Presumably, only specific degrees of expression of TBX3 and interactors result in mesenchymal commitment and differentiation. The TBX3 partner (or partners) driving this differentiation program remain to be identified. Previously, it has been shown that TBX3 cooperates with c-Myc to induce the transformation of sarcoma-initiating cells [31]. Interestingly, c-Myc is the critical effector of *Akt/FBXW7ΔF*-dependent cholangiocarcinogenesis [31]. In addition, synergistic activity of TBX3, c-Myc, and AKT has been revealed in rhabdomyosarcoma [31]. Thus, the TBX3/c-Myc and/or Akt cooperation may also be responsible for osteosarcoma-like lesions occurrence in *Akt/FBXW7ΔF/TBX3* mice. Intriguingly, human hepatoblastomas with an osteoid component are well-known in clinical practice [31], and cases of liver cirrhosis evolving into osteosarcomas have been previously described [31]. Thus, it would be critical to determine whether TBX3 plays a role in these tumor subsets. Future research should be devoted to addressing this issue.

Because strategies aimed at reactivating TBX3 in iCCA lesions might be harmful due to the risk of inducing mesenchymal tumors, TBX3 downstream targets whose inactivation is detrimental to biliary tumorigenesis should be unraveled. In this regard, using RNA-seq and qPCR screening, we identified *MAD2L1* as a potential gene suppressed by TBX3 in all iCCA cell lines tested. TBX3 and MAD2L1 expression appeared to correlate inversely in human and mouse iCCA lesions. Subsequent experiments showed that overexpression of *MAD2L1* increased the proliferation of iCCA cell lines in vitro, and knockout of *Mad2l1* markedly reduced the tumor burden and prolonged the survival of mice in vivo. Therefore, MAD2L1 might be a valuable therapeutic target in iCCA. The protein product of *MAD2L1* is part of the mitotic spindle assembly checkpoint that halts anaphase until proper chromosomal alignment occurs [32]. The expression of *MAD2L1* was studied in resected iCCA specimens and correlated with tumor size, pathological grade, clinical stage, and inversely with overall survival [32]. Therefore, targeting MAD2L1 might be an attractive strategy against iCCA. Currently, no small molecules against MAD2L1 have been reported, and additional efforts are required to develop new drugs targeting MAD2L1. In addition, gene-based therapies, such as CRISPR-based gene deletion or microRNA-based silencing, could be explored as innovative strategies against MAD2L1.

Chemotherapy, consisting of Gemcitabine and Cisplatin-based therapies, is still the mainstream treatment option for advanced, unresectable, or recurrent iCCA [33]. However, most patients do not respond to the chemotherapy [33]. In the current study, we uncovered the expression and functions of the TBX3/MAD2L1 pathway in regulating cholangiocarcinogenesis. As most iCCA samples are TBX3 low and MAD2L1 high, it is tempting to hypothesize that these tumors might be less responsive to the chemotherapy due to the more aggressive tumor cell phenotypes. Additional studies, both in vitro and in vivo, will be required to address how the TBX3/MAD2L1 pathway regulates drug responsiveness in iCCA.

### Supplementary information

Supplementary materials

Supplementary Table 3

Western blot original data

## Data Availability

All data generated or analyzed during this study are included in this published article and its supplementary information files.
