# Supplementary material for: Overexpression of TBX3 suppresses tumorigenesis in experimental and human cholangiocarcinoma
Source: Cell Death Dis. 2024 Jun 22;15(6):441. doi: 10.1038/s41419-024-06839-8 (PMC11193761; doi:10.1038/s41419-024-06839-8)

Supplementary data for "Overexpression of TBX3 suppresses tumorigenesis in experimental and human cholangiocarcinoma"

Authors: Shanshan Deng[1,2,†], Xinjun Lu[3,†], Xue Wang[1, †], Binyong Liang[2], Hongwei Xu[2], Doris Yang[1], Guofei Cui[1,2], Andrew Yonemura[1], Honor Paine[2], Yi Zhou[2], Yi Zhang[4], Maria Maddalena Simile[5], Francesco Urigo[6], Matthias Evert[6], Diego F. Calvisi[6], Benjamin L. Green[1,*], and Xin Chen[1,2,*]

Affiliations: [1]Cancer Biology Program, University of Hawai'i Cancer Center, University of Hawai'i, Honolulu, Hawaii, USA.[2] Department of Bioengineering and Therapeutic Sciences and Liver Center, University of California, San Francisco, California, USA. [3]Department of Biliary-Pancreatic Surgery, Sun Yat-sen Memorial Hospital, Sun Yat-sen University, Guangzhou, China. [4]School of Pharmacy and Bioengineering, Chongqing University of Technology, Chongqing, 400054, China. [5]Department of Medicine, Surgery, and Pharmacy, Division of Experimental Pathology and Oncology, University of Sassari, 07100 Sassari, Italy; [6]Institute of Pathology, University of Regensburg, Regensburg, Germany.

[†] S. Deng, X. Lu, and X. Wang contributed equally to this article.

* X. Chen and B.L. Green are co-corresponding authors.

Supplementary Table 1. Sequences of sgMAD2L1.

Genes	sgRNA forward sequences, 5’-3’	sgRNA reverse sequences, 5’-3’
sgMAD2L1 (human)	CACCGTATTTCTGCACTCGAGTAA	AAACTTACTCGAGTGCAGAAATAC
sgMAD2L1 (mouse)	CACCGATATTTCTGCACTCTAGTAA	AAACTTACTAGAGTGCAGAAATATC

Supplementary Table 2. Sequences of quantitative real-time PCR primers.

Genes	Forward primer sequences, 5'-3'	Reverse primer sequences, 5'-3'
18s rRNA	CGGCTACCACATCCAAGGAA	GCTGGAATTACCGCGGCT
CALM2 (human)	CAACAAAGGAATTGGGAACTG	TGTCATCACATGGCGAAGTT
CALM2 (mouse)	ACGGGGATGGGACAATAACAA	TGCTGCACTAATATAGCCATTGC
HSH2D (human)	TGTGAACTTGTCGTCACTCTTG	GAGGGGCTTTTGAGAGATGTG
HSH2D (mouse)	GGACTTTTCTTATCCGAGTCAGC	TAGGCTTTTGTTGGTGGAAGG
IDUA (human)	CAGGAGATACATCGGTAGGTACG	TCATGGAGACGTTGTCAAAGTC
IDUA (mouse)	GCTGACCAGTACGACCTTAGT	TACGGCACCTATGTAGGCAAG
MAD2L1 (human)	ATCACAGCTACGGTGACATTTC	GCGGACTTCCTCAGAATTGGT
MAD2L1 (mouse)	GTGGCCGAGTTTTTCTCATTTG	AGGTGAGTCCATATTTCTGCACT
PFDN4 (human)	AGTGGAATCAATTCAGCGAGTG	GCTTCAAGGTTTATGTTGCTCCC
PFDN4 (mouse)	CAGATCGGAGACGTTTTCATCA	ACTCTGGACTCTAAGGCATCAAT
RGS17 (human)	CAGAGGCCCAACAACACCTG	TGTGGGTCTTCCCGCATTTT
RGS17 (mouse)	GCAGTCACAAAATGAAGGAACAC	GGGATCTCCCCGAAGAGTCT
SUB1 (human)	GGTGAGACTTCGAGAGCCCT	GCGAACACTAACGTACCTCATTT
SUB1 (mouse)	AGCAAGCGGTTCCAGAGAAG	TCCCGAACACTGACATATCTCA
TBX3 (human)	GTGTCTCGGGCCTGGATTC	ACGTGTAGGGGTAAGGGAACA
TBX3 (mouse)	CAGGCAGCCTTCAACTGCTT	GGACACAGATCTTTGAGGTTGGA
ZDHHC13 (human)	ACCCCACTCTTATTGATGGAGA	TGTCTGCCCATTTACATCTGTC
ZDHHC13 (mouse)	TCGCAGTGCAGGAATCACAG	GGCAGCCCAGTGAAGAAGA

Supplementary Table 3. logFPKM data of parental or TBX-overexpressing KKU-156 cells.

Supplementary Table 4. Potential transcriptional targets regulated by TBX3.

	JASPAR	UCSC (Track Jaspar)	GeneCards
RGS17	TBX3,relative score=0.94	TBX3 binding (p<0.0001)	-
IDUA	TBX3,relative score=0.96	TBX3 binding (p<0.0001)	TBX3 binding
PFDN4	TBX3,relative score=0.95	TBX3 binding (p<0.0001)	TBX3 binding
SUB1	TBX3,relative score=0.97	TBX3 binding (p<0.0001)	TBX3 binding
CALM2	TBX3,relative score=0.94	TBX3 binding (p<0.0001)	TBX3 binding
ZDHHC13	TBX3,relative score=0.96	TBX3 binding (p<0.0001)	TBX3 binding
MAD2L1	TBX3,relative score=0.94	TBX3 binding (p<0.0001)	-
HSH2D	TBX3,relative score=0.98	TBX3 binding (p<0.00001) (3 binding positions)	TBX3 binding

Supplementary Figure 1. Development of mesenchymal/osteoblastic-like lesions in *Akt/FBXW7ΔF/TBX3* mice. (A) In addition to small intrahepatic cholangiocarcinomas, 3 of 10 *Akt/FBXW7ΔF/TBX3* mice developed mesenchymal lesions in the liver. (B) At higher magnification, these lesions were characterized by spindle/sarcomatoid cells immersed in an abundant, densely-packed matrix. (C) Calcifications (indicated by the dotted circle) were often detected in these lesions. (D) Furthermore, these lesions exhibited the focal deposition of an osteoid substance (Osteoid), suggesting the presence of osteosarcoma-like lesions. (E) The osteoid substance was surrounded by multinucleated cells (indicated by arrows), reminiscent of osteoclasts. The lesions expressed the injected oncogenes (indicated by immunoreactivity for activated/phosphorylated form of Akt, or p-Akt, FBXW7, and TBX3), implying their origin from the injected genes. (I, L) In addition, the positive immunoreactivity of the mesenchymal cells for the SATB2 transcription factor (I) and the RANK ligand (RANKL) (L) further substantiated the presumable osteoid nature of these lesions. Also, they showed elevated immunopositivity for the proliferation marker Ki67 (inset in L), suggesting their malignant nature. Original magnifications: 20x in (A); 200x in B, D, F, G, H, I, L; 400x in C, E, and inset. Scale bar: 1000 µm in (A); 100 µm in B, D, F, G, H, I, and L; 50 µm in C and E.

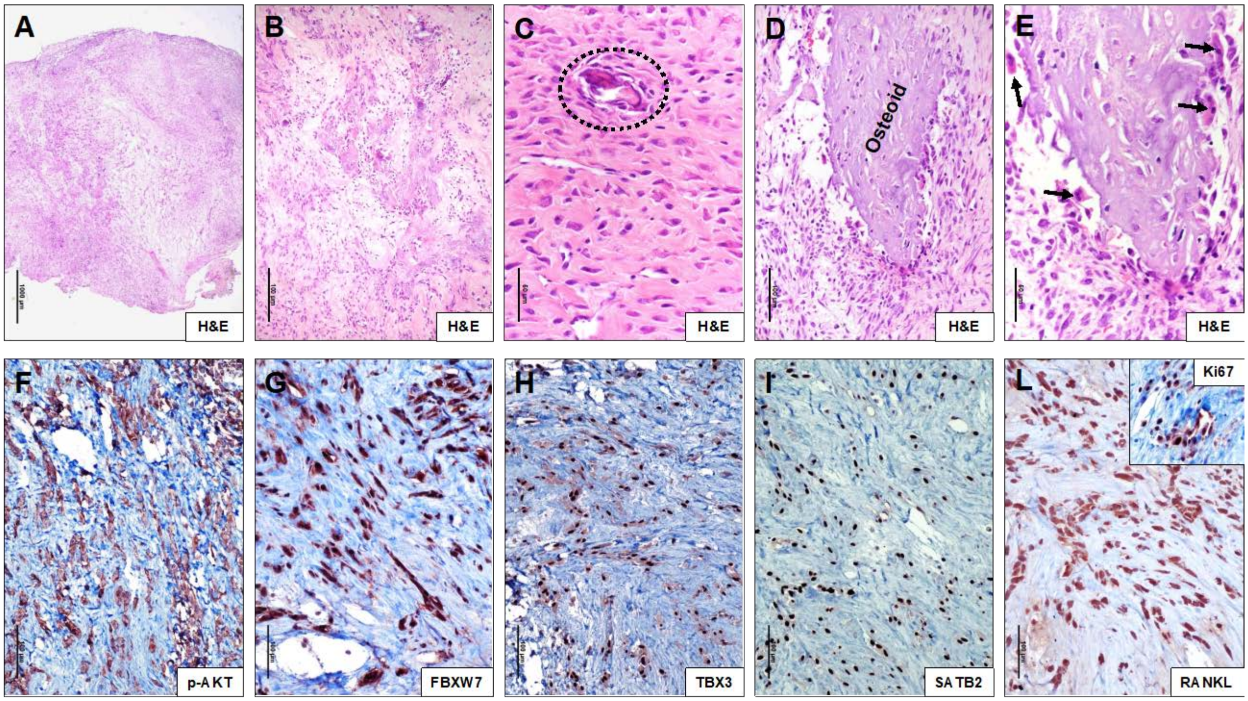

Supplementary Figure 2. RNA-seq analysis in TBX3-overexpressing KKU-156 cells. (A) The gene expression profile heatmap of the parental and TBX3-overexpressing KKU-156 cells. (B) KEGG analysis of the downregulated pathways.

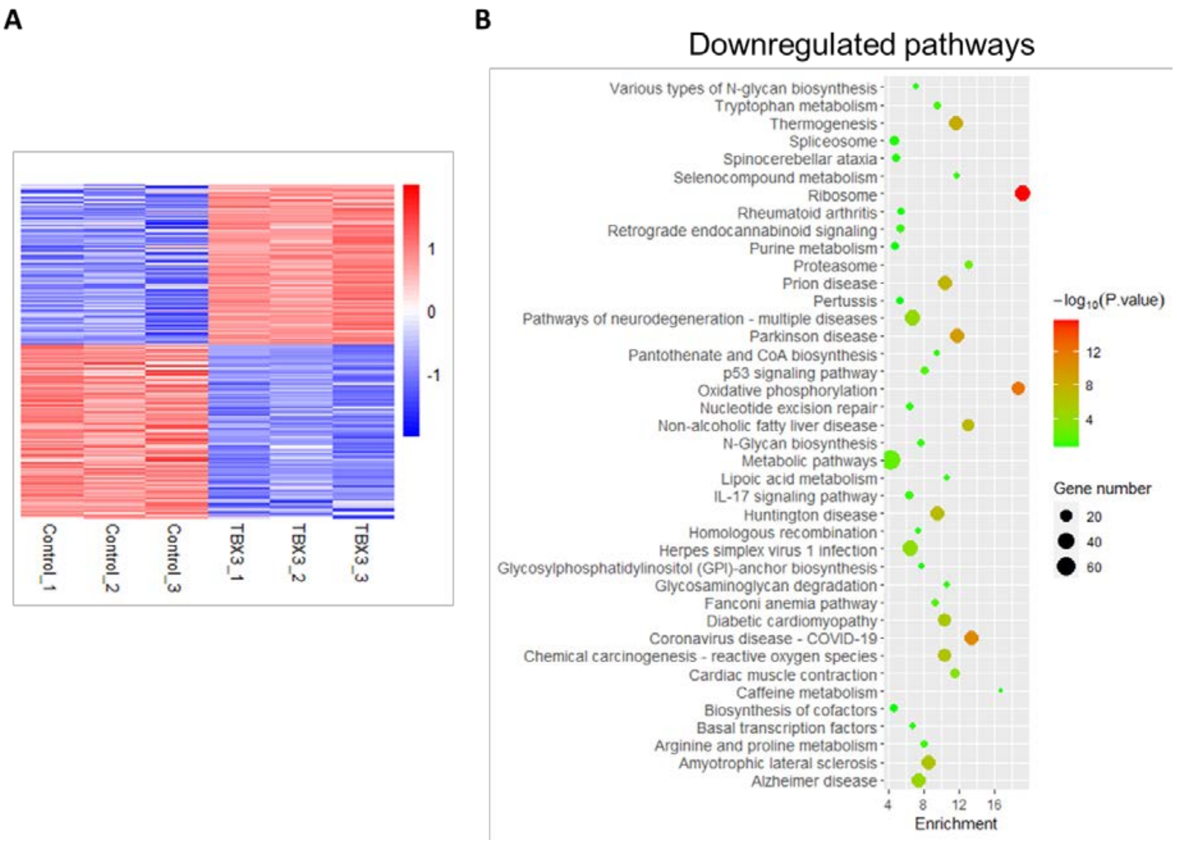

Supplementary Figure 3. Validation by q-RT-PCR of putative TBX3 target genes identified by RNA-Seq in parental and TBX3-overexpressing iCCA cell lines and mouse livers. (A) Parental and TBX3-overexpressing RBE cells. (B) Parental and TBX3-overexpressing KKU-156 cells. (C) Liver tissues from *Akt/FBXW7△F/pT3* and *Akt/FBXW7△F/TBX3* mice. TBX3 was used as a positive control.

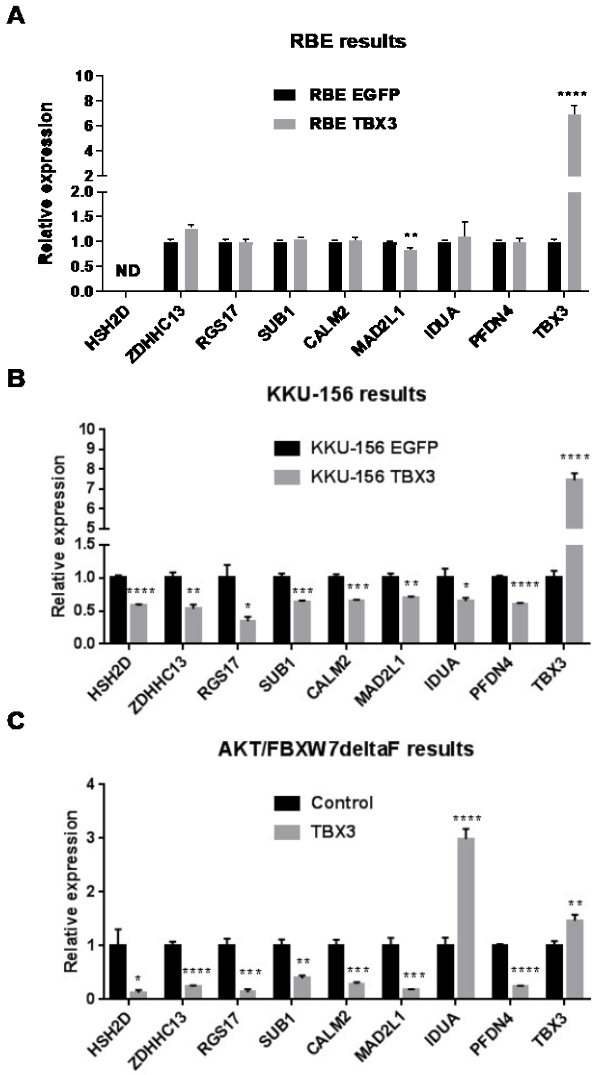

Supplementary Figure 4. Overexpression of *MAD2L1* promotes the proliferation of iCCA *in vitro*, as assessed by colony formation assay.

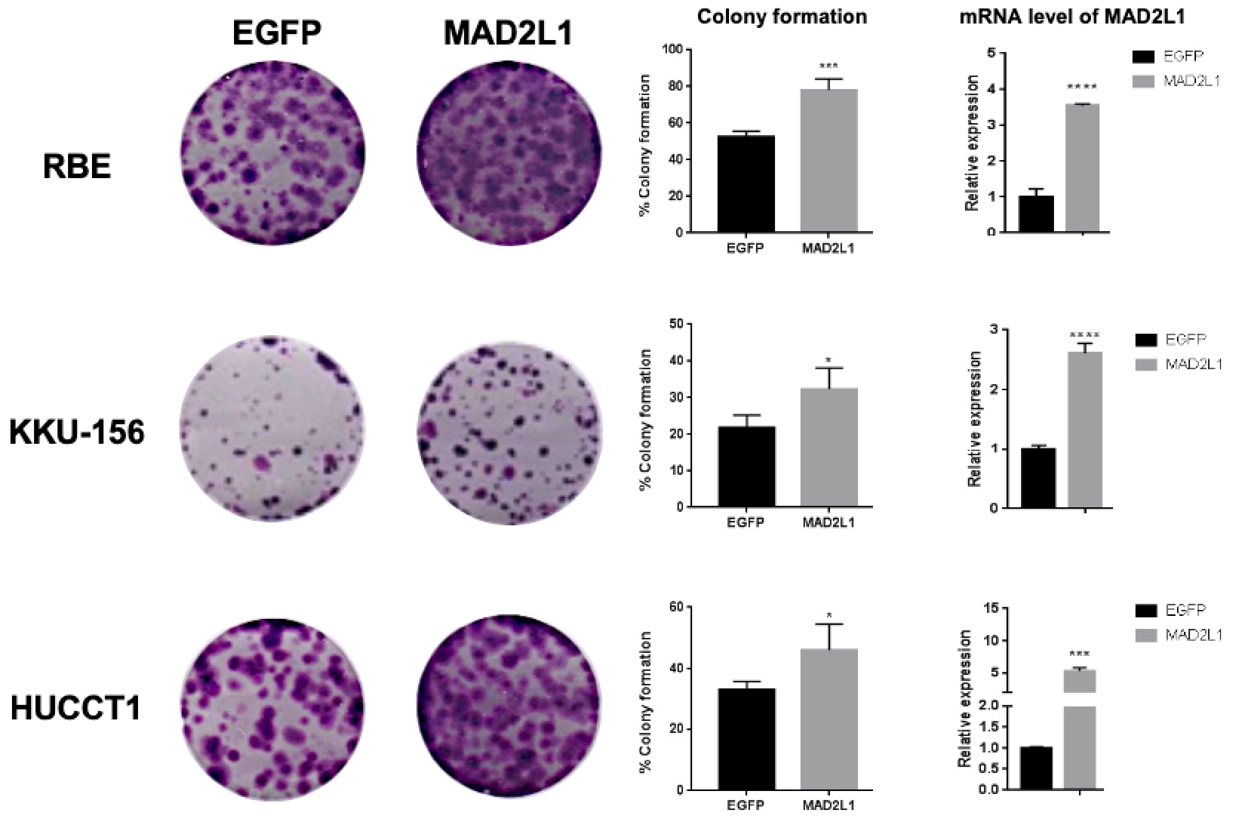

Supplementary Figure 5. Knocking down of *MAD2L1* inhibits the growth of iCCA *in vitro*. (A) Colony formation assay of RBE and HUCCT1 iCCA cells with or without *MAD2L1* silencing. (B) Western blot analysis of MAD2L1 in the samples collected in (A). β-ACTIN was used as a loading control. (C) Standard PCR and Sanger sequencing were used to validate the editing of *MAD2L1* by sequencing its genomic region. Simply, we designed a pair of PCR primers to amplify the knockout target sequence from the DNA samples of each group. The amplified product's size was approximately 500 bp, with the gRNA targeting site positioned at its center. After gel purification, we performed Sanger sequencing on the purified PCR product, using either the forward or reverse primer. Subsequently, we analyzed the sequencing outcomes using TIDE. The overall efficiency of the gRNA should exceed 30%. The bars to the left of "column 0" represent the percentage of deleted sequence in the DNA, while the bars to the right of "column 0" represent the percentage of inserted sequence in the DNA.

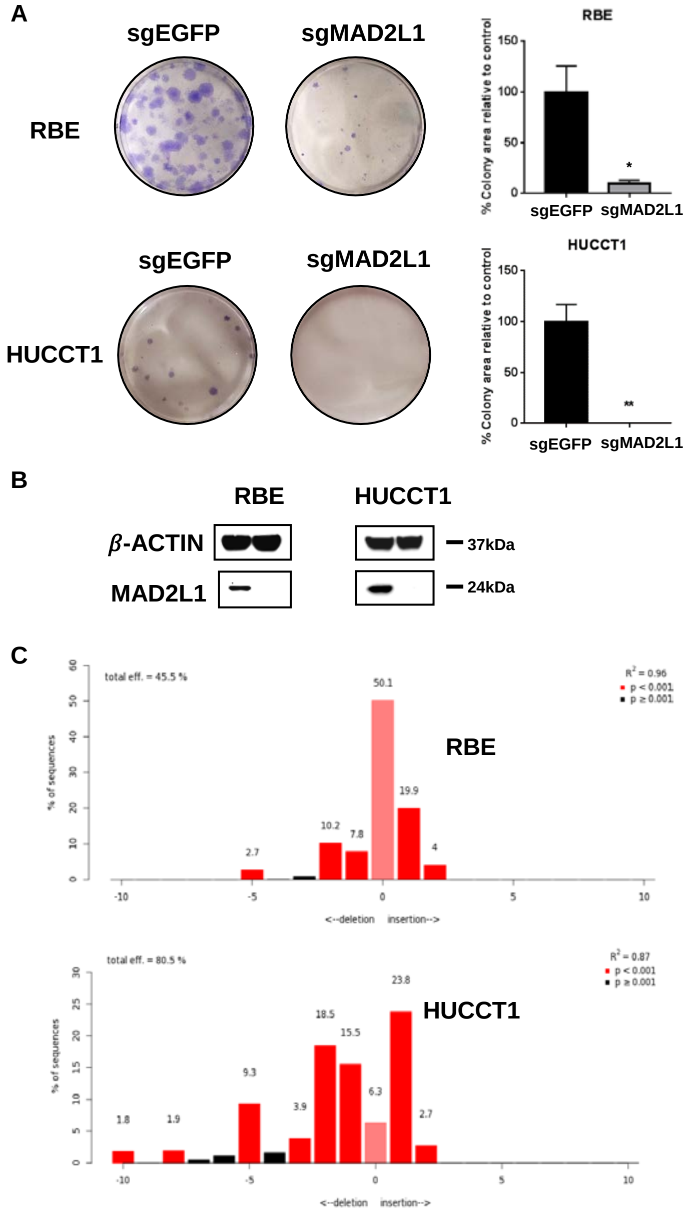

Supplementary Figure 6. Knockdown efficiency of the *sgMad2l1* construct in mice. The total knockdown efficiency is approximately 85%.

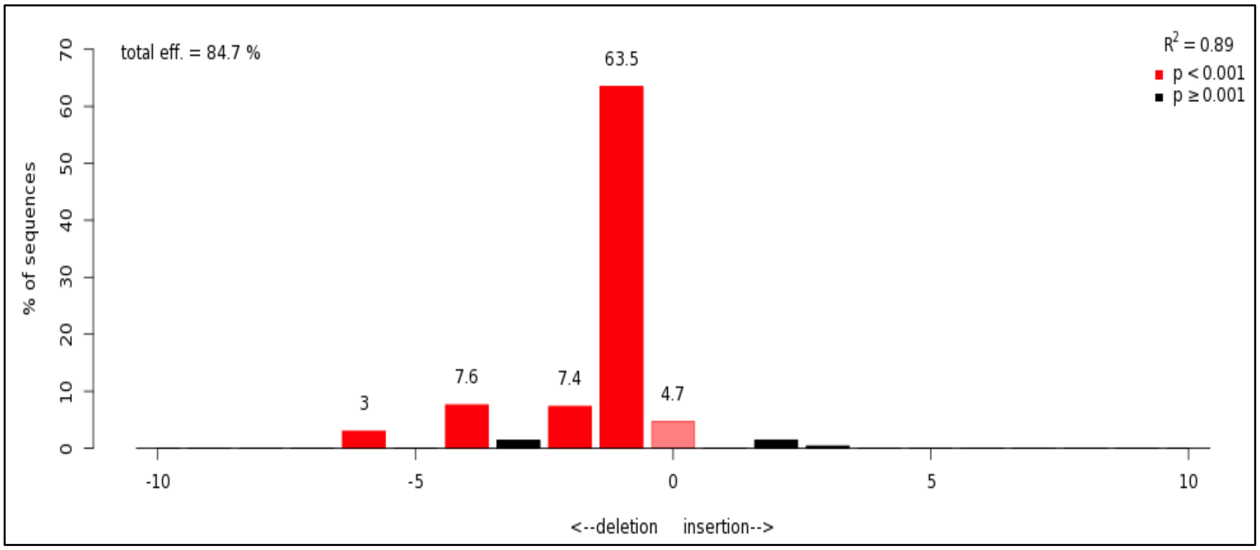

Supplementary Figure 7. Knocking down *Mad2l1* significantly delays tumor development in the Akt/NICD iCCA model. (A) Study design. FVB/N mice were hydrodynamically injected with *Akt/NICD/sgEGFP* or *Akt/NICD/sgMad2l1* plasmids. Mice were sacrificed on week 5 post-injection. (B) Representative gross liver images, H&E stained liver images, and the liver weight of mice in each group are depicted. (C) qRT-PCR analysis of *Tbx3* and *Mad2l1* mRNA expression in *Akt/NICD/sgEGFP* or *Akt/NICD/sgMad2l1* liver tissues.

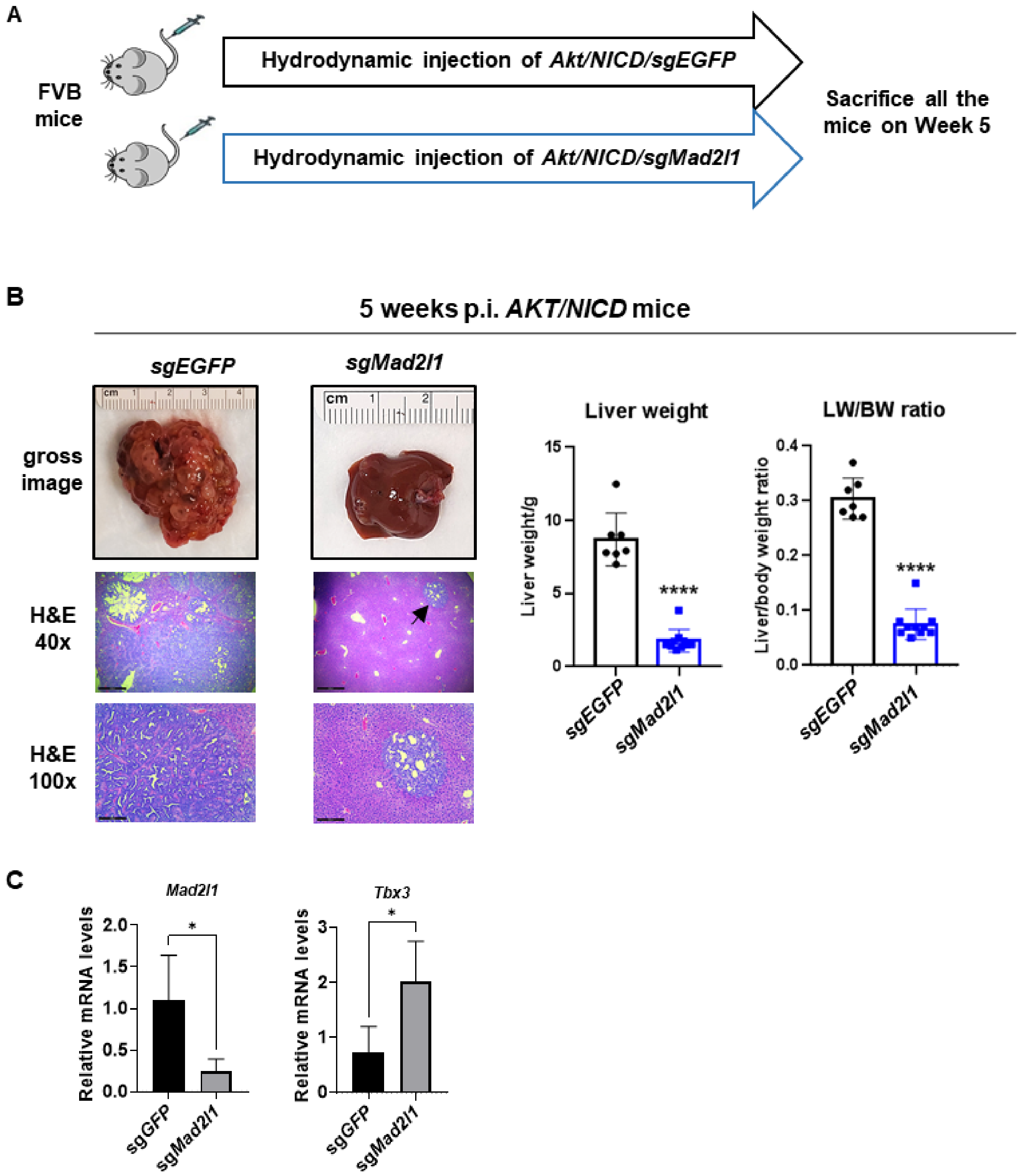

Supplementary Figure 8. KEGG pathway analysis of TBX3 co-expressed genes in NCI iCCA database.

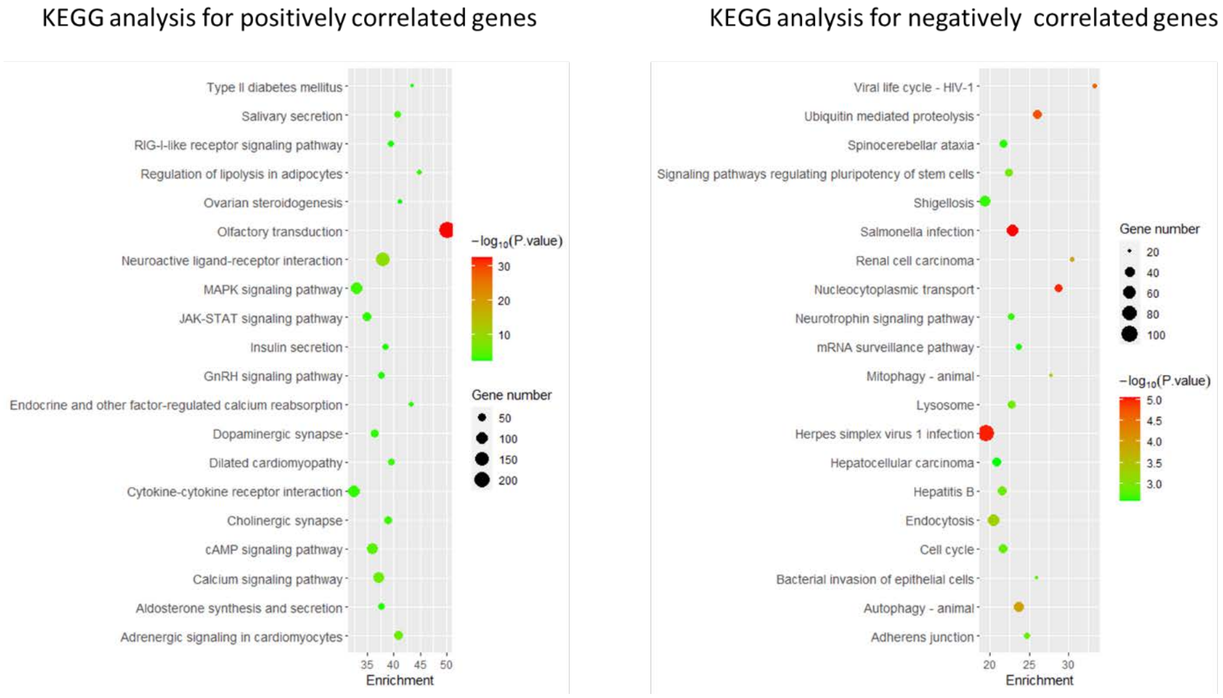

Supplementary Figure 9: The correlation between TBX3 and CCNE1, CCNB1, PLK1, CDK4 and PCNA, respectively.

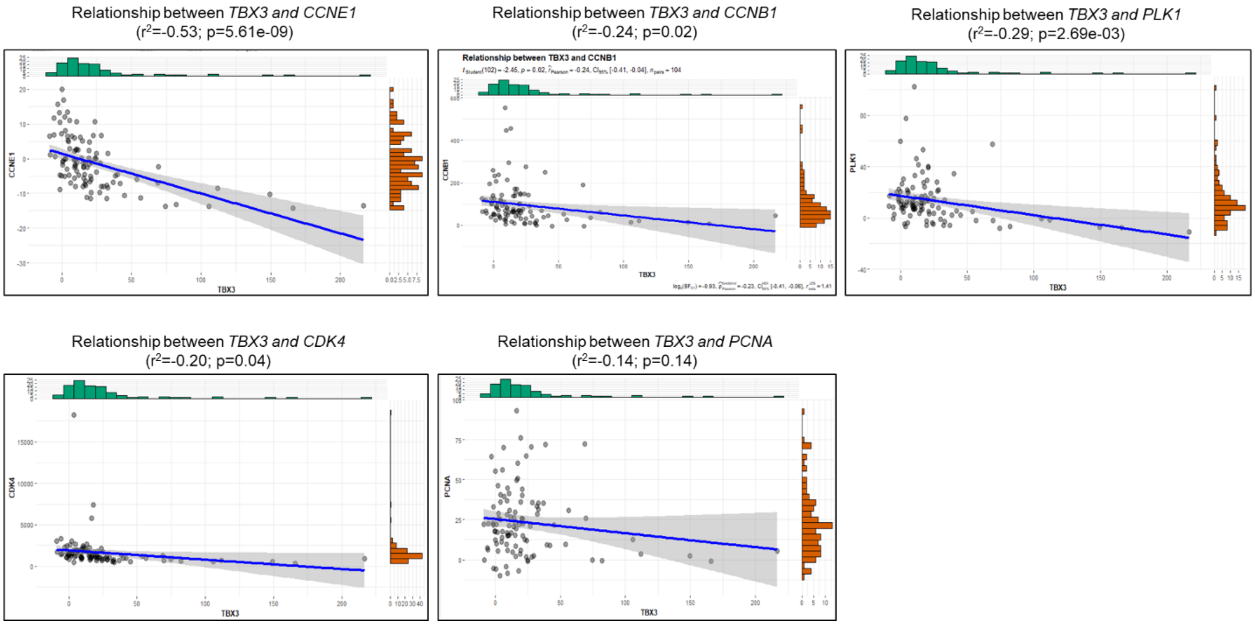

Supplementary Figure 10. Representative immunohistochemical patterns of TBX3 and MAD2L1 proteins in human surrounding non-tumorous liver tissue. Non-neoplastic hepatocytes and cholangiocytes (indicated by arrows and positive for the biliary marker CK19) exhibited moderate nuclear staining for TBX3. In contrast, cytoplasmic immunoreactivity for MAD2L1 characterized non-tumorous hepatocytes, whereas no MAD2L1 immunolabeling was detected in cholangiocytes. Abbreviation: H&E, hematoxylin and eosin staining. Original magnification: 200× in all panels. Scale bar: 100 µm in all panels.

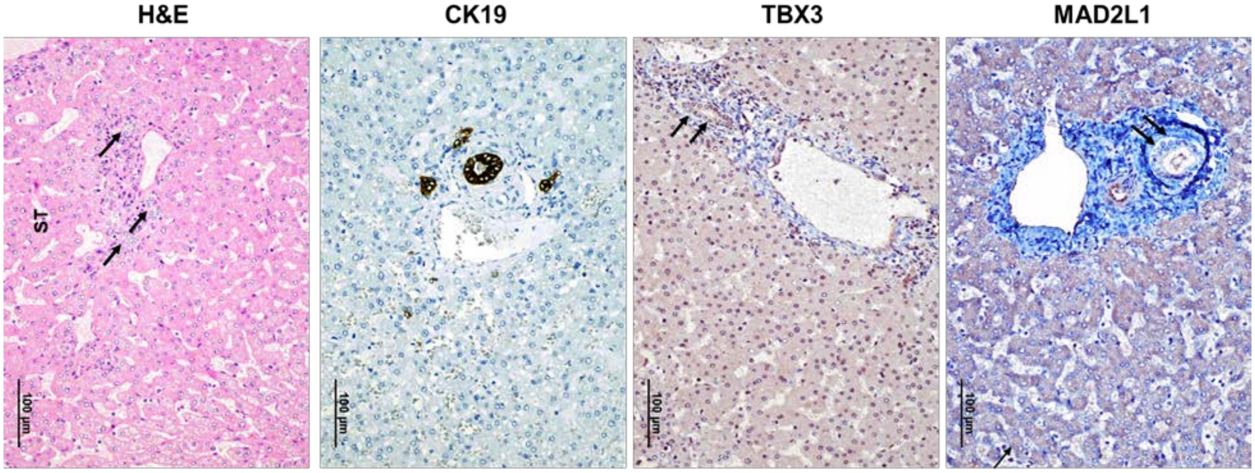

Supplement: Supplementary file 1 — Supplementary materials [file 41419_2024_6839_MOESM1_ESM.pdf]
