# Supplementary material for: Overexpression of TBX3 suppresses tumorigenesis in experimental and human cholangiocarcinoma
Source: Cell Death Dis. 2024 Jun 22;15(6):441. doi: 10.1038/s41419-024-06839-8 (PMC11193761; doi:10.1038/s41419-024-06839-8)
Supplement: Supplementary file 3 — Western blot original data [file 41419_2024_6839_MOESM3_ESM.pptx]

## Slide 1
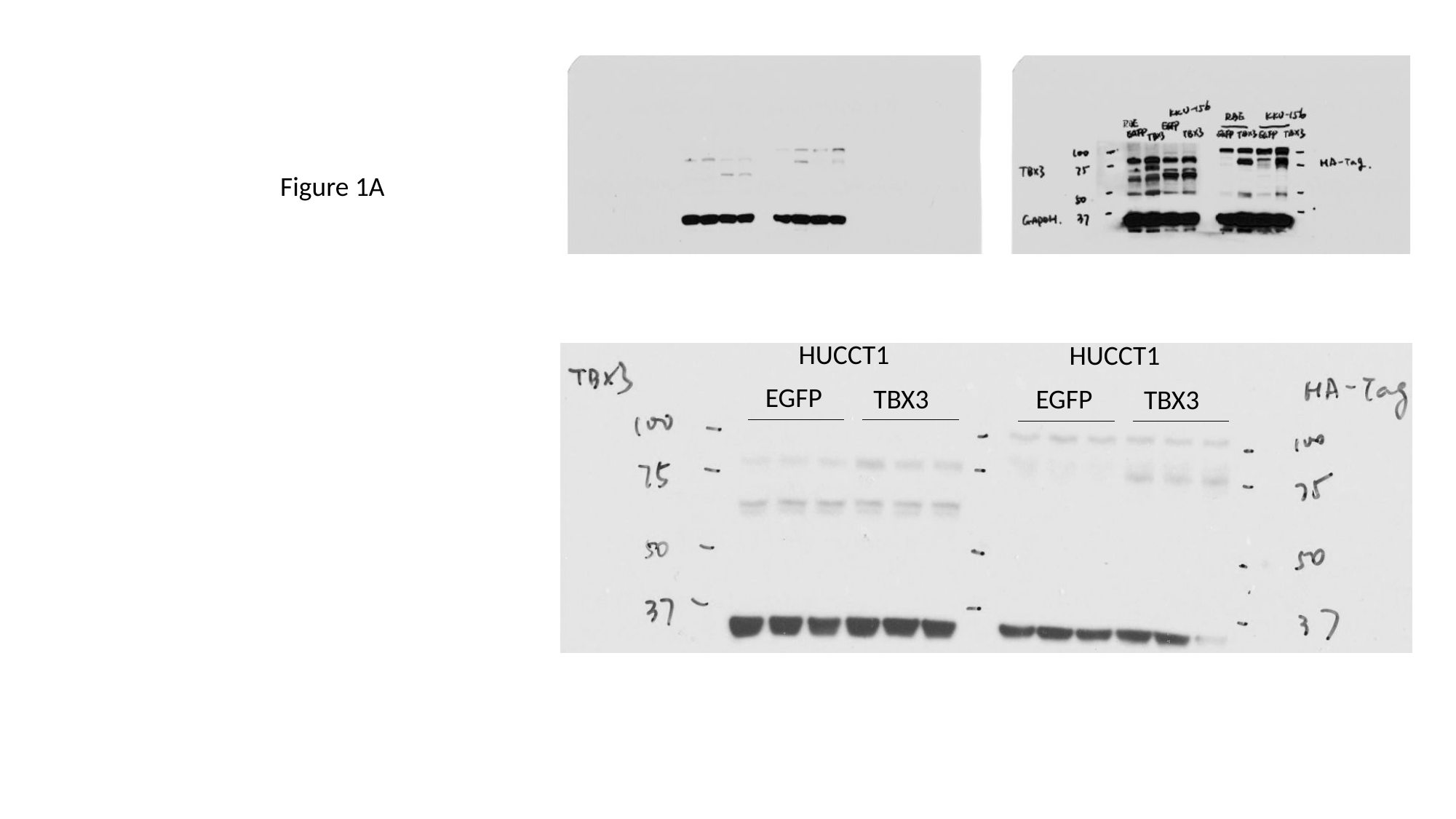

Figure 1A
HUCCT1
HUCCT1
EGFP
EGFP
TBX3
TBX3

## Slide 2
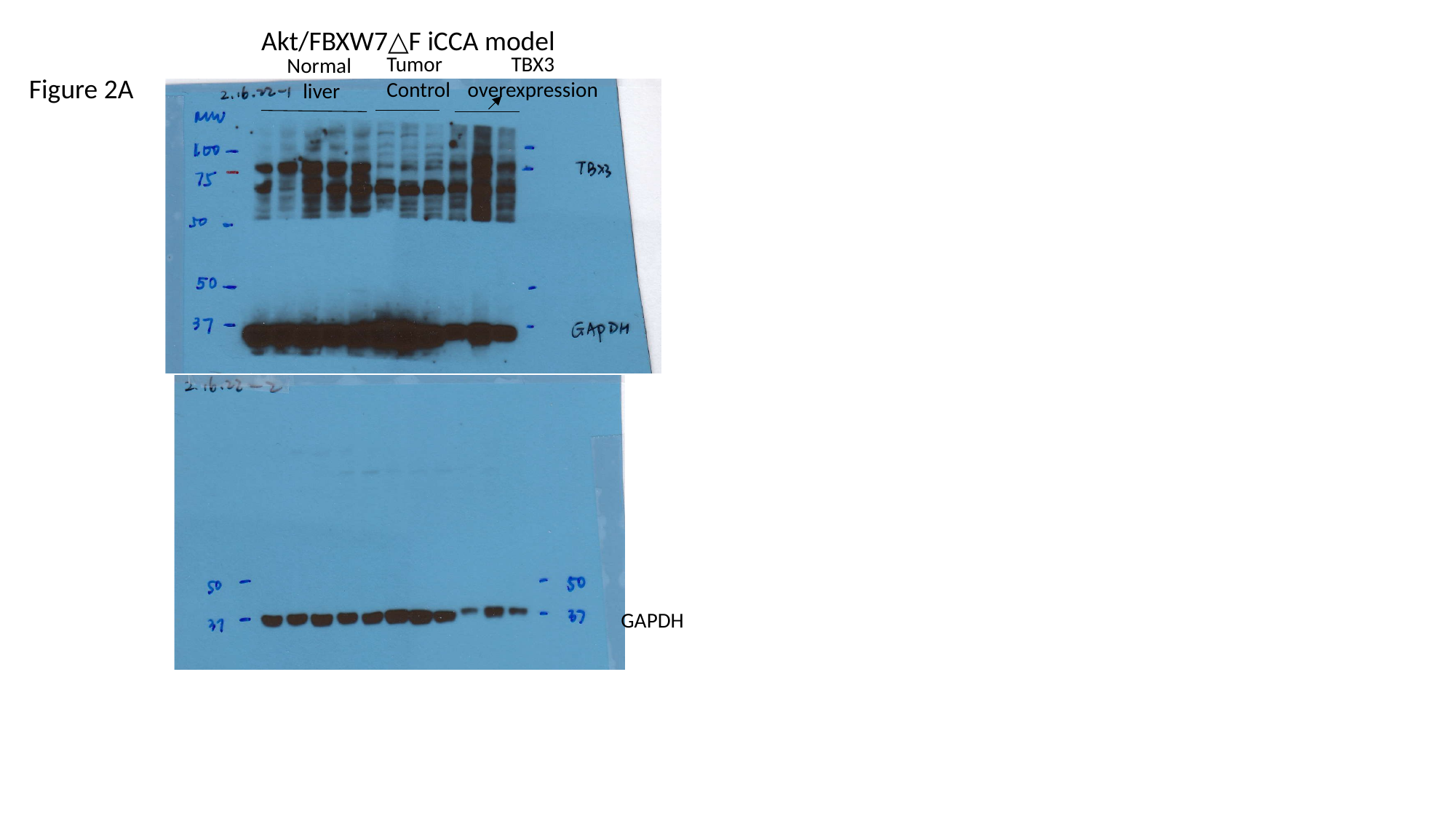

Akt/FBXW7△F iCCA model
Tumor
Control
TBX3
overexpression
Normal
liver
Figure 2A
GAPDH

## Slide 3
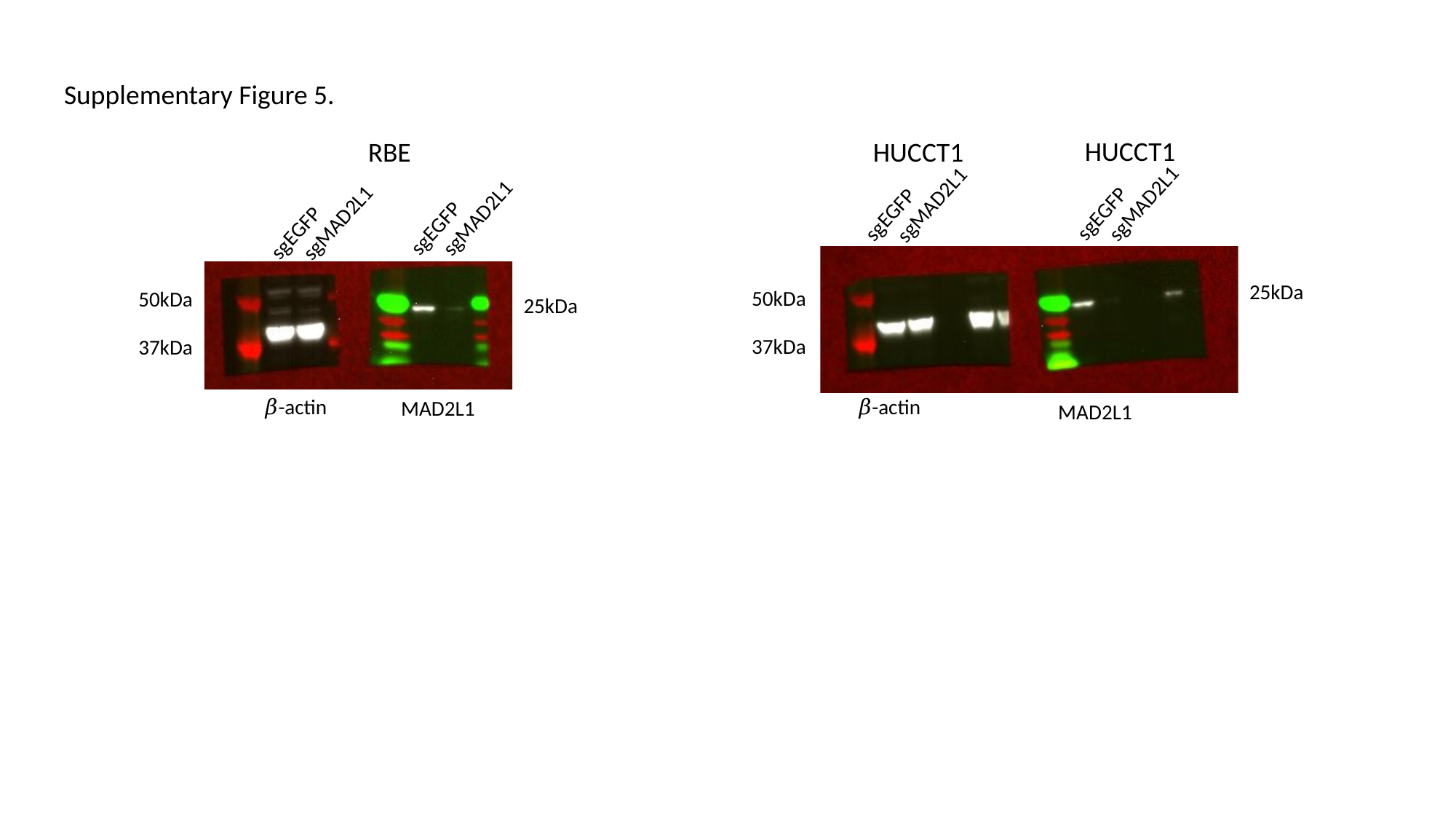

Supplementary Figure 5.
HUCCT1
RBE
HUCCT1
sgMAD2L1
sgMAD2L1
sgEGFP
sgEGFP
sgMAD2L1
sgMAD2L1
sgEGFP
sgEGFP
25kDa
50kDa
50kDa
25kDa
37kDa
37kDa
𝛽-actin
𝛽-actin
MAD2L1
MAD2L1
